# Feed binders to improve the quality of Atlantic salmon (*Salmo salar*) feed and faeces

**DOI:** 10.7717/peerj.21291

**Published:** 2026-06-22

**Authors:** André Sture Bogevik, Tor Andreas Samuelsen, Andre Meriac, René Alvestad, Turid Synnøve Aas

**Affiliations:** 1 Department of Nutrition and Feed Technology, Nofima, Fyllingsdalen, Vestland, Norway; 2 Department of Production Biology, Nofima, Tromsø, Troms, Norway; 3 Department of Nutrition and Feed Technology, Nofima, Sunndalsøra, Møre og Romsdal, Norway

**Keywords:** Atlantic salmon, Digestibility, Faecal collection efficiency, Feed binders, Solid waste removal

## Abstract

Binders are commonly incorporated into aquafeeds to improve pellet durability during transport and handling, as well as to enhance water stability. These functional properties may also affect faecal characteristics, which are critical for efficient removal of solid waste in aquaculture systems. Two control feeds were formulated: one fish-meal-based feed (FM, 60% FM) and one plant-meals-based-feed (PM, with 15% FM). An additional 14 PM-based feeds were produced by including one of the following binders; guar gum, xanthan gum, lignin sulfonate, potato starch, pea starch, alginate or gelatine—at 2 or 5%. Atlantic salmon postsmolt were fed these feeds in two preliminary trials: Trial 1 compared the PM control with feeds containing 2% binders, and Trial 2 compared FM control with feeds containing 5% binders. In Trial 1, faecal material could only be collected from fish fed 2% guar gum, whereas faeces were collected from all groups in Trial 2. Based on these results, the FM control, PM control and feeds containing 2% guar gum, xanthan gum and alginate were selected for a final trial (Trial 3). Significantly more faecal material was collected from salmon fed guar gum, followed in descending order by FM control > alginate > xanthan gum > PM control. Guar gum also resulted in significantly lower nutrient digestibility and, together with xanthan gum, increased intestinal viscosity and decreased faecal dry matter content compared to the control feeds (PM < FM). Faeces from fish fed the FM control contained less carbohydrates and more ash than faeces from PM-based feeds, which may have contributed to differences in faecal stability and should be investigated further.

## Introduction

Feed binders are used in aquafeed extrusion to produce pellets with sufficient durability for bulk storage, transport, and delivery through pneumatic feeding systems. In Atlantic salmon (*Salmo salar*) feeds, starch-rich ingredients such as wheat, pea, and bean meals are commonly included because they contribute to predictable pellet quality and are partially digestible (Aas, Åsgård & Ytrestøyl, 2022). During extrusion, water, steam and mechanical shear transform the formulated ingredients into expanded extrudates, which are subsequently dried and oil-coated in a vacuum system. The processability of the ingredients and the physical properties of the final pellets depend on their physicochemical and rheological characteristics, as well as on the type of plasticizers and binders used and the specific process configuration (Sørensen, 2012; Samuelsen, Mjøs & Oterhals, 2013, 2014; Samuelsen & Oterhals, 2016).

Pellet stability can be enhanced through the inclusion of polysaccharide binders such as guar gum, alginate, or starch (Pearce, Daggett & Robinson, 2002). Starch remains the most commonly used binder in salmon feeds, and its binding capacity varies with the amylose-to-amylopectin ratio of the source material. Many polysaccharides have gelling properties, and alginate in particular forms thermally stable gels in the presence of divalent cations. Incorporating alginate into biodegradable films has been shown to improve mechanical strength and water resistance (Zhao et al., 2017). Protein hydrolysates can act as plasticizers, improving extruder cooking efficiency and water stability of the final pellets (Samuelsen & Oterhals, 2016; Ahmad et al., 2019). However, despite their widespread use in feed manufacturing, the effects of these binders on faecal stability and faeces collection efficiency in Atlantic salmon are not well documented. Developing improved binder and plasticizer strategies has the potential to enhance pellet strength, faecal stability and thereby increase the efficiency of solid-waste removal in land-based salmon production systems.

The expansion of land-based Atlantic salmon farming has heightened the need for effective management of uneaten feed and faecal waste. Both must be sufficient intact to be efficiently removed by filtration systems. However, faeces are often poorly removed in practice. In a commercial smolt facility equipped with advanced filtration, only 30–40% of the estimated waste was recovered as sludge (Aas & Åsgård, 2019), likely due to small particles and dissolved material that escape filter screens. As uneaten feed and faeces travel through long pipe systems before reaching the filters, the fragile nature of salmon faeces leads to rapid disintegration into fine particles that are difficult to retain. Feed pellets generally maintain their structural integrity more effectively and may constitute a substantial proportion (up to 50%) of the sludge dry matters (Aas, Ytrestøyl & Berge, 2016; Aas & Åsgård, 2019). Loss of uneaten feed and faeces represents a discharge of valuable nutrients into the environment. In recirculating aquaculture systems (RAS), ineffective solid-waste removal can further promote heterotrophic bacterial growth, elevate microparticle concentrations, impair biofilter performance, and increase the risk of off-flavour development, opportunistic pathogen blooms, or hydrogen sulphide formation (Timmons, Guerdat & Vinci, 2018).

Faeces must possess adequate firmness and be transported as little as possible before entering filtration units to allow efficient collection. Salmon faeces form pellet-like structures with inherently fragile consistency, and their water content, viscosity, and durability are strongly influenced by dietary composition. Certain ingredients are known to induce diarrhoea in salmon (*e.g.*, soybean meal), and higher water content is often seen in faecal content of salmon fed feeds with high inclusion of plant proteins (Baeverfjord & Krogdahl, 1996). Conversely, inherent non-starch polysaccharides may function as a natural binder, improving solid-waste removal and reduce organic loading on biofilters (Meriac et al., 2014). Previous research has shown that adding 0.3% guar gum to rainbow trout feed increases faecal viscosity and resistance to disintegration in water (Brinker & Friedrich, 2012; Welker, Overturf & Barrows, 2020). Replacement of native starch with gelatinized starch has reduced the proportion of dissolved faecal material in effluent water (Amirkolaie, Verreth & Schrama, 2006). Other studies demonstrate that binder inclusion (guar gum, alginate, starch) increases faecal stability by enhancing the elastic properties of digesta (Brinker, 2007, 2009). High inclusion levels, however, may slow gastrointestinal passage and negatively affect feed intake, digestibility and growth (Storebakken, 1985). While several studies indicate beneficial effects of binders on faecal integrity, including some recent work in Atlantic salmon (Prakash et al., 2025), a systematic evaluation of different binder types and inclusion levels with respect to feed and faecal collection efficiency in this species is still lacking.

The present study employed a newly developed tank-outlet collection system that enables separation of uneaten pellets from faeces. Using this system, binders were incorporated at 2 and 5% inclusion levels to evaluate their effects on feed and faecal collection efficiency. Additional assessments included feed technical quality, feed intake, faecal output, faecal properties, and nutrient digestibility in Atlantic salmon. This approach provides a systematic framework for evaluating binder effects on solid-waste management and addresses an important knowledge gap in land-based salmon production.

## Materials and Methods

The study was conducted at the Research Station for Sustainable Aquaculture, Nofima, Sunndalsøra, Norway. All experimental procedures complied with the Norwegian Animal Welfare Act and EU Directive 2010/63/EU on the protection of animals used for scientific purposes. Procedures were performed by FELASA-accredited personnel (functions A–D). The experimental protocol was approved according to the regulation of the Norwegian Food Safety Authority (Mattilsynet).

### Selection of binders

Seven binders were selected for evaluation: guar gum (Alimenta AS, Oslo, Norway), xanthan gum (Alimenta AS, Oslo, Norway), lignin sulfonate (Borregaard, Sarpsborg, Norway), potato starch (Alimenta AS, Oslo, Norway), pea starch (AM Nutrition AS, Stavanger, Norway), alginate (Sigma-Aldrich, St. Louis, MO, USA) and gelatine (Alimenta AS, Oslo, Norway).

### Feeds

The experimental feeds were formulated using ingredients commonly incorporated in commercial Atlantic salmon feeds (Aas, Åsgård & Ytrestøyl, 2022). Two control feeds were produced; a high fish meal (FM) feed containing 600 g kg^−1^ fish meal, and a high plant meal (PM) feed containing 150 g kg^−1^ fish meal (Table 1). Fourteen additional feeds were produced by replacing 2 and 5% of the PM feed formulation with each binder. All feed were produced at the Aquafeed Technology Centre (Nofima, Bergen, Norway).

**Table 1:** Formulation (g kg ^−1^) of the fish meal (FM) control feeds and plant meal (PM) control feed.

Feed formulation	FM	PM
Fish meal	600.0	150.0
Soy protein concentrate (SPC)		200.0
Corn gluten		50.0
Wheat	119.4	79.4
Wheat gluten		140.0
Faba beans		60.0
Fish oil	120.0	130.0
Rapeseed oil	120.0	130.0
Choline chloride	5.0	5.0
Lecithin	5.0	5.0
L-Lysin		10.0
Astaxanthin	0.5	0.5
Mineral premix	5.0	5.0
Vitamin premix	5.0	5.0
Mono sodium phosphate (MSP)	20.0	20.0
Calcium carbonate (CaCO_3_)		10.0
Yttrium(III)oxide (Y_2_O_3_)	0.1	0.1
Chemical composition		
Dry matter (g/100g)	93.0	94.7
Crude protein (g/100g)	42.6	40.6
Total fat (g/100g)	28.6	27.0
Gross energy (kJ/g)	23.3	23.9

### Fish trials, pre-trials (Trials 1 and 2)

Two preliminary trials were conducted to evaluate the impact of 2 and 5% binder inclusion on faecal quality. In Trial 1 (15 days), salmon were fed PM control or feeds containing 2% binder. In Trial 2 (14 days), fish were fed FM control or feeds containing 5% binder. Fish in one tank were given the control feeds, while the feeds with the respective binders were given to two replicate tanks.

Atlantic salmon postsmolt (374 ± 3 g) were randomly allocated to 15 tanks (1,900 L), with 27 fish per tank, on 3 November 2022. A total of 405 fish were required to obtain sufficient faecal material for analysis, corresponding to a stocking density of 5.3 kg m^−3^ at the start of the experiment. The tanks were supplied with flow-through seawater at 12.1 ± 0.1 °C and maintained under continuous light and feeding.

**Figure 1:**
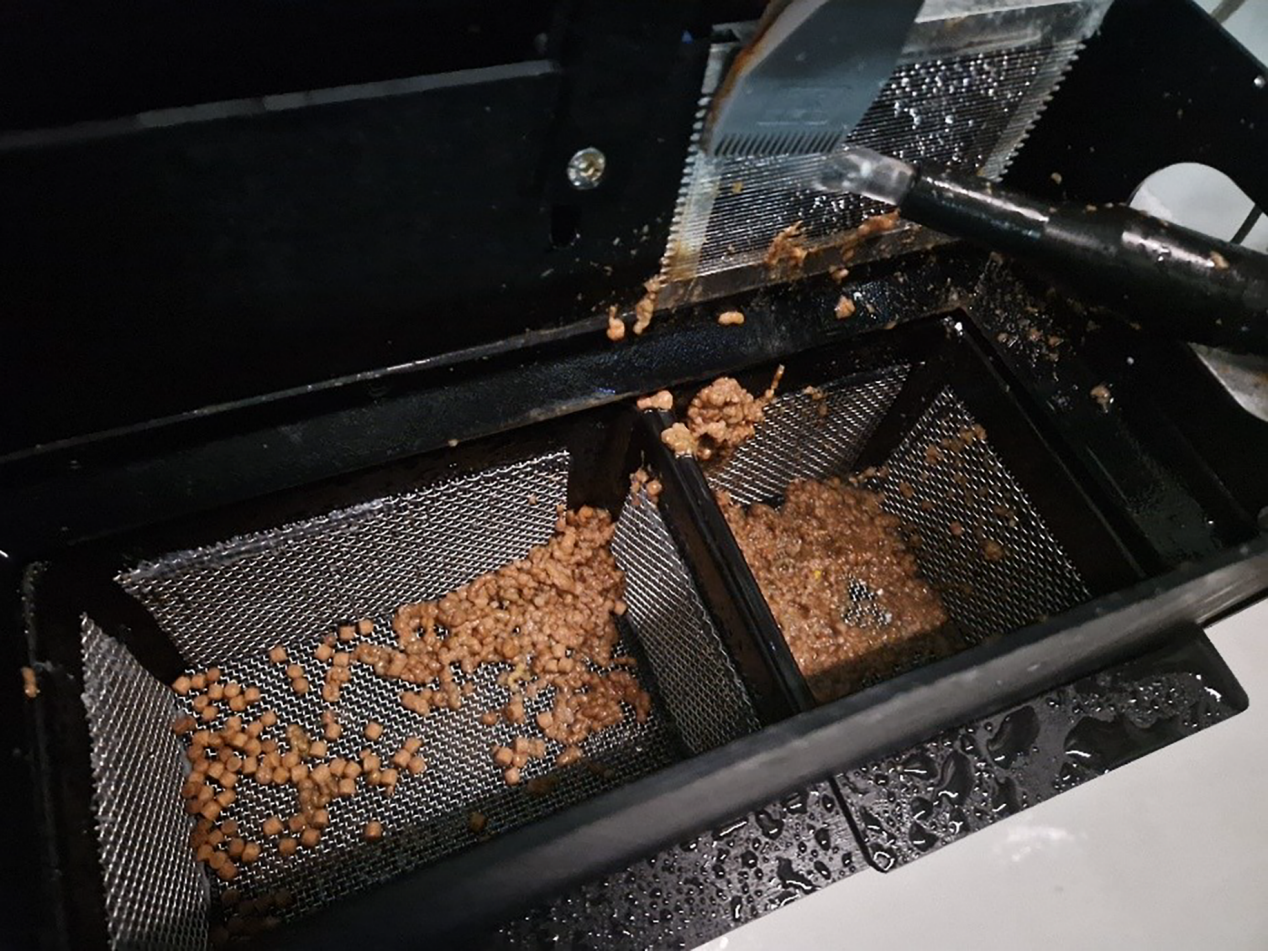
Spillbox device for collection of uneaten feed (left) and faeces in different chambers.

Each tank was fitted with a custom-design Nofima “Spillbox” (patent pending) that separated uneaten feed and faeces into separate chambers (Fig. 1). Feeds were assigned randomly using a spreadsheet algorithm. The experimental periods were; Trial 1: 3–18 November (PM control + 2% binder feeds) and Trial 2: 18 November – 3 December (FM control + 5% binder feeds). Feed delivery and fish health were monitored daily, and no mortality or clinical signs of disease were observed. Uneaten feed and faeces collected in the Spillbox were quantified daily to calculate feed intake and assess binder effects on faecal characteristics.

Results from Trial 1 and 2 are shown in Table 2. Feed intake did not differ significantly between dietary treatments in either trial (ANOVA; *P* < 0.05). Feed containing guar gum at both 2% and 5% significantly increased faecal collection relative to all other feeds. In Trial 1, the 2% guar gum feed yield more than 20-fold greater faecal recovery than other treatments. In Trial 2, the 5% xanthan gum feed resulted in approximately five-fold greater faecal collection compared with lignin sulfonate, potato starch and pea starch (Tukey *post hoc* test; *P* < 0.05). The FM control feed also produced high faecal recovery.

**Table 2 table-2:** Calculated feed intake and measured Spillbox content of faeces (as is) in the pre-trials with Atlantic salmon fed feeds with 2 and 5% binders, respectively, for a period of 14 days.

Trial 1	Feed intake (g/day)	Faeces collected (g)	Trial 2	Feed intake (g/day)	Faeces collected (g)
Control PM	72^a^	19^a^	Control FM	136^a^	299^abc^
Guar gum	79 ± 6^a^	912 ± 69^b^	Guar gum	96 ± 15^a^	478 ± 43^c^
Xanthan gum	80 ± 14^a^	45 ± 6^a^	Xanthan gum	118 ± 10^a^	336 ± 122^bc^
Lignin sulfonate	66 ± 12^a^	12 ± 11^a^	Lignin sulfonate	107 ± 7^a^	43 ± 38^a^
Potato starch	80 ± 3^a^	19 ± 16^a^	Potato starch	111 ± 14^a^	72 ± 27^a^
Pea starch	74 ± 20^a^	17 ± 18^a^	Pea starch	127 ± 1^a^	55 ± 14^a^
Alginate	70 ± 10^a^	17 ± 3^a^	Alginate	104 ± 7^a^	101 ± 52^ab^
Gelatine	73 ± 9^a^	12 ± 2^a^	Gelatine	123 ± 6^a^	109 ± 48^ab^

**Note:**

Mean ± standard deviation, *n* = 2 except control feeds where *n* = 1. Statistics by ANOVA followed by Tukey *post hoc* test. Superscripts showing different letters are significantly different (*P* < 0.05).

### Main fish trial (Trial 3), experimental design, sampling, and chemical analysis

Based on pre-trial results, guar gum and xanthan gum were selected for further evaluation due to their strong effects on faecal collection. Alginate was included due to its relevance being a sustainable sidestream from seaweed and kelp production in Norway. Gelatine, despite similar performance to alginate, was excluded due to lower long-term relevance and consumer acceptance, compared to alginate in future sustainable feed formulations. Lignin sulphonate, pea starch, and potato starch feeds showed consistently poor faecal recovery and were not included in Trial 3.

Feeds containing 2% binder were chosen for the final trial as this inclusion level is most relevant commercially and avoid excessive dilution of nutrient-dense ingredients. The FM control, PM control, and the three selected binder feeds were fed to salmon in triplicate tanks for 2 weeks (3–15 December 2022). The same fish from Trial 1 and 2 were retained to avoid handling-related effects; body weight were not measured prior to the start of Trial 3.

At termination, all fish were euthanized with an overdose of Finquel MS-222 (3 g L^−1^ tricaine methanesulfonate, Scan-Vacc, Hvam, Norway) following EU Directive 2010/63/EU and FELASA guidelines. Ten fish per tank were measured for body weight, length, gutted weight, and sex. Gastrointestinal (GI) content was weighted separately for the stomach, proximal gut (anterior gut with pyloric caeca), mid gut and distal gut (posterior part of the intestine with increased diameter) as described in Bogevik et al. (2021). Total GI content was expressed as a percentage of body weight, and content of each gut segment was expressed as percentage of total GI content to describe digesta distribution along the tract. Remaining fish were euthanized and faeces were collected by stripping according to the method by Austreng (1978). Distal gut content obtained *via* dissection and stripped faeces were pooled per tank and stored at −20 °C prior to digestibility analysis.

Crude protein in the feeds and samples of freeze-dried GI content was analysed using the Kjeldahl method (N × 6.25; ISO 5983-1997). Moisture (ISO 6496-1999) and ash (ISO 5984-2002) were determined gravimetrically after drying preweighed samples in porcelain cups at 103 ± 1 °C for 4.5 h followed by incineration of the dried samples at 550 ± 20 °C for 16 h. Lipid content was analysed using the European Commission (2009) method with acid hydrolysis. Yttrium was measured by inductively coupled plasma atomic emission spectroscopy (ISO 11885-1996).

### Feed mix viscosity and physical pellet quality

Viscosity profiles of the PM control, FM control and three binder diets (2% inclusion) were measured using a ViscoQuick instrument (Brabender® GmbH & Co., Duisburg, Germany). Diets were ground using a Retsch ZM-1 centrifugal mill (Retsch GmbH, Haan, Germany) with a ring sieve aperture of 0.5 mm. A 25.0 g dry matter sample was added distilled water giving a total weight of 115.0 g. The following instrument profile was used; hold at 25 °C in 5 min; ramp up to 95 °C from 5 to 10 min; hold at 95 °C in 7 min, cool-down to 25 °C from 17 to 22 min; hold at 25 °C in 8 min. The paddle was run at 60 rpm for 10 s to disperse the sample and then at a constant speed of 250 rpm the rest of the test period. The following parameters were determined: Cold viscosity (maximum viscosity at 25 °C); Peak viscosity (maximum viscosity at 95 °C); Hold viscosity (minimum viscosity at 95 °C) and Final viscosity (maximum viscosity after cool-down to 25 °C). Reported values were the mean of duplicate measurements.

Pellet hardness was measured as the peak breaking force in Newton and was conducted on coated laying pellets by use of a texture analyzer (TA.XTplusC, Stable Micro Systems Ltd., Surrey, UK) as described in Samuelsen & Oterhals (2016). Individual pellets were measured, and reported as mean of 20 pellet.

Pellet water stability index (WSI) was determined according to the method by Baeverfjord et al. (2006). Briefly, 20 g of coated pellets were placed in steel-mesh baskets immersed in 1,000 ml glass beaker filled with 500 ml tap water. The beakers were incubated in a thermostat-controlled water bath at 23 °C and agitated at 160 times/min for 120 min. Remaining dry matter was quantified, with results expressed as the mean of triplicate measurements.

### Calculations and statistics

All calculation were performed in Microsoft Excel. Apparent digestibility coefficient (ADC) of nutrients and energy in the test feeds was calculated from the following formula: ADC = 100 – 100 × [Yd/Yf] × [Nf/Nd] where d is feed, f is faeces, Y is yttrium concentration and N is nutrient concentration. Total faecal matter produced per tank during the trial was calculated based on total feed intake and percentage of indigested dry matter calculated in stripped faeces. And furthermore, divided between amount of dry faeces collected in the Spillbox and remaining not collected and released into the waste system. Results are presented at mean ± standard deviation with tank as the statistical unit, except for biometic measures on fish that were measured in individual fish. Homogeneity of variance in results between groups were tested by Levene’s Test, followed by log transformation of significant values (*P* < 0.05). The significant difference between means were analysed by one-way analysis of variance (ANOVA), followed by Tukey’s multiple range test to determined individual differences between groups. Statistical analyses were performed using STATISTICA v13.5 (StatSoft, Inc. Tulsa, OK, USA).

## Results

### Feed mix viscosity and technical physical feed quality

**Figure 2:**
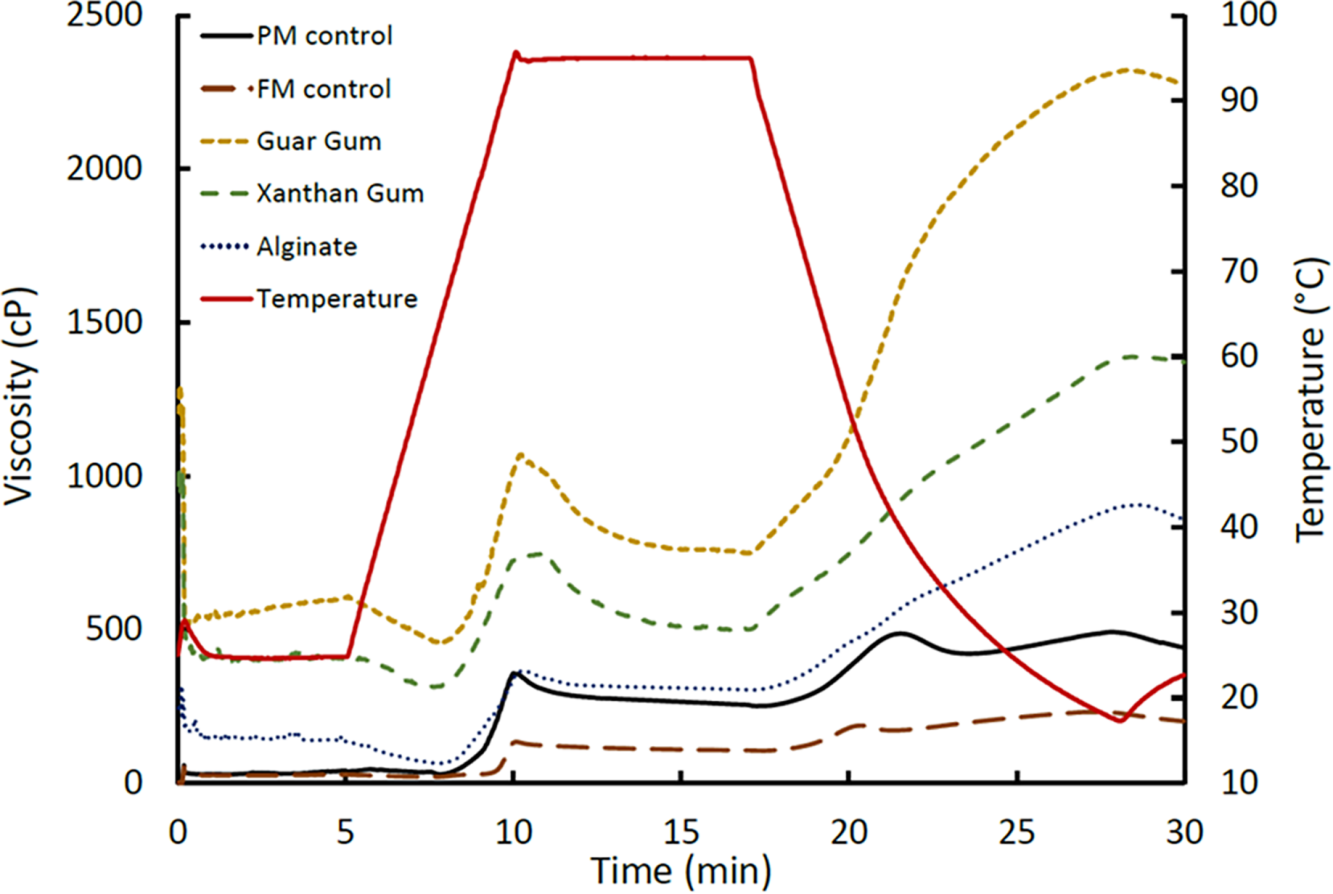
ViscoQuick viscosity profiles for the plant meal (PM) and fishmeal (FM) feed mixes and feed mixes containing three selected binders at 2% inclusion levels. Viscosity parameters can be found in Table S1.

Significant differences in cold, peak, hold and final viscosity were observed for the selected feed mixes (Fig. 2, Table S1). The FM control feed mix consistently displayed the lowest viscosity values, followed by the PM control, alginate, and xanthan, whereas the guar gum feed mix produced a markedly greater increase in viscosity compared to all the other binders.

Pellet hardness also differed significantly among diets: the FM control feed had the lowest hardness (27.5 N), whereas the PM control feed had the highest (59.9 N; Table S2). Water-solubility index (WSI) values ranged from 83 to 88%, and although these values different statistically, all feeds were classified as having high WSI. Except for the FM control feed, which showed both the lowest hardness and WSI, there were poor correlation between the two analyses in this study.

### Feed and faeces mass-balance in feeding trial with postsmolt salmon

**Figure 3:**
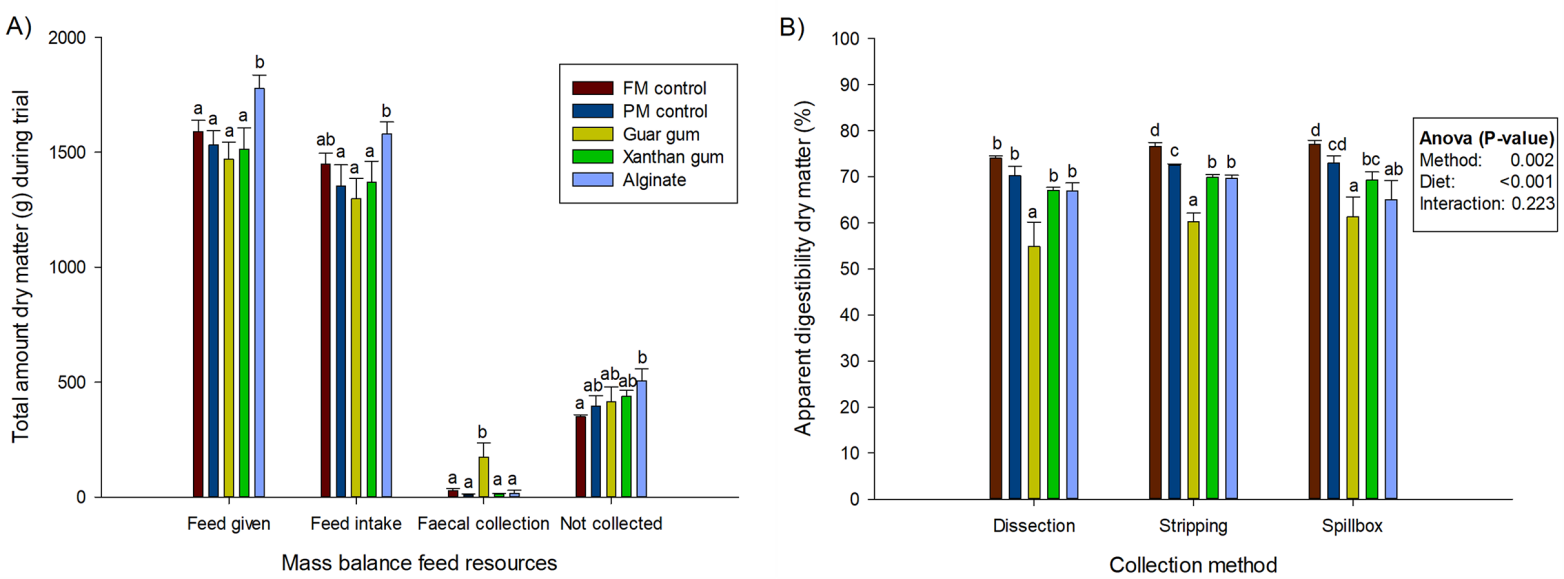
Mass balance of feed and faeces; (A) Calculated dry matter (DM) of feed given, feed intake, collected and not collected faeces from fish fed control feeds and test feeds with selected binders. (B) Dry matter digestibility calculated in faecal content collec. Mean ± standard deviation, *n* = 3. Statistics by ANOVA followed by Tukey *post hoc* test. Data for faecal collected and not collected, and DM digestibility of stripped content were log-transformed prior to analysis due to variance heterogeneity (Levene’s test). Superscripts showing different letters are significantly different (*P* < 0.05) between dietary groups, and collection method for digestibility calculations.

At the end of Trial 3, salmon had a mean final weight of 579 g. Mean daily feed intake per tank ranged from 108 to 148 g/day, corresponding to 0.7–1.0% of biomass based on final weight. Of the total feed distributed, 87–92% was consumed, resulting in an estimated dry matter intake of approximately 1,400 g per tank. Total dry matter intake was significantly higher in salmon fed the feed containing 2% alginate compared to those fed the PM control and feeds containing 2% guar gum or 2% xanthan gum (Tukey *post hoc*, *P* < 0.05; Fig. 3A).

Dry faecal matter collected in the Spillbox was significantly higher for salmon fed guar gum compared with both control feeds and feeds containing 2% xanthan gum or 2% alginate (Tukey *post hoc*; *P* < 0.05; Fig. 3A). Dry matter digestibility in salmon fed guar gum (54–61%) was significantly lower than in all other groups (64–77%), irrespective of collection method. Stripped faeces showed dietary differences similar to those observed in the Spillbox, with the highest digestibility in the FM control, followed by the PM control, intermediate values for xanthan gum and alginate, and the lowest values guar gum (Tukey *post hoc* test; *P* < 0.05; Fig. 3B).

Across diets, dry matter digestibility was highest in faeces collected from the Spillbox, intermediate in stripped samples, and lowest in distal gut contents collected by dissection (ANOVA; *P* < 0.05). The reduced digestibility in the guar gum group resulted in significantly greater dry faecal matter produced compared to both control feeds (Tukey *post hoc*, *P* < 0.05). Although salmon fed guar gum produced more faecal matter that entered the Spillbox, the amount of uncollected material did not differ from the other groups. In contrast, higher feed intake combined with low Spillbox collection in salmon fed alginate resulted in a significant greater amount of uncollected faecal matter released to the system relative to the other feeds (Fig. 3).

### Gastrointestinal response to feeds with binders

The GI contents corresponded to [Table table-2]approximately 10% of body weight (Table 3). Although variations was high within groups, salmon fed the guar gum feed had significantly greater GI content (11% of the body weight) compared to those fed the alginate feed (8% of the body weight) (ANOVA; *P* = 0.014). The greatest proportions of content were found in the proximal gut (43–51%) and mid gut (35–48%). Salmon fed alginate had higher stomach and distal gut content than those fed the other feeds. Guar gum fed salmon showed a distribution similar to the alginate group in the proximal gut, but with significantly lower proportions than the other groups. In addition, guar gum produced significantly greater mid gut content than all other groups (ANOVA; *P* < 0.05; Tables 3 and S3).

**Table 3:** Total GI content (as is) of fish body weight and relative amount of content in different GI segments of dissected Atlantic salmon (10 individuals per tank) fed either control feeds with FM, PM or 2% inclusion of guar gum, xanthan gum or alginate in triplicate tanks for a period of 14 days.

Feeds	Total GI (% of BW)	Stomach (% of total)	Proximal (% of total)	Midgut (% of total)	Distal (% of total)
Control FM	9.5 ± 2.8^ab^	1.3 ± 1.2^a^	48.9 ± 3.5^b^	44.3 ± 2.5^bc^	5.5 ± 2.3^a^
Control PM	10.3 ± 4.2^ab^	2.0 ± 1.6^a^	50.6 ± 4.9^b^	41.7 ± 3.9^b^	7.1 ± 3.4^a^
Guar gum	11.1 ± 3.7^b^	1.8 ± 1.4^a^	44.3 ± 3.8^a^	47.7 ± 4.1^c^	6.3 ± 2.9^a^
Xanthan gum	10.6 ± 3.7^ab^	1.7 ± 1.5^a^	49.0 ± 3.7^b^	42.6 ± 2.5^b^	6.7 ± 3.1^a^
Alginate	7.9 ± 5.2^a^	4.5 ± 4.3^b^	43.3 ± 9.0^a^	35.1 ± 10.7^a^	17.1 ± 16.1^b^

**Note:**

Mean ± standard deviation, *n* = 3. Statistics by ANOVA followed by Tukey *post hoc* test. Superscripts showing different letters are significantly different (*P* < 0.05) between dietary groups.

**Figure 4:**
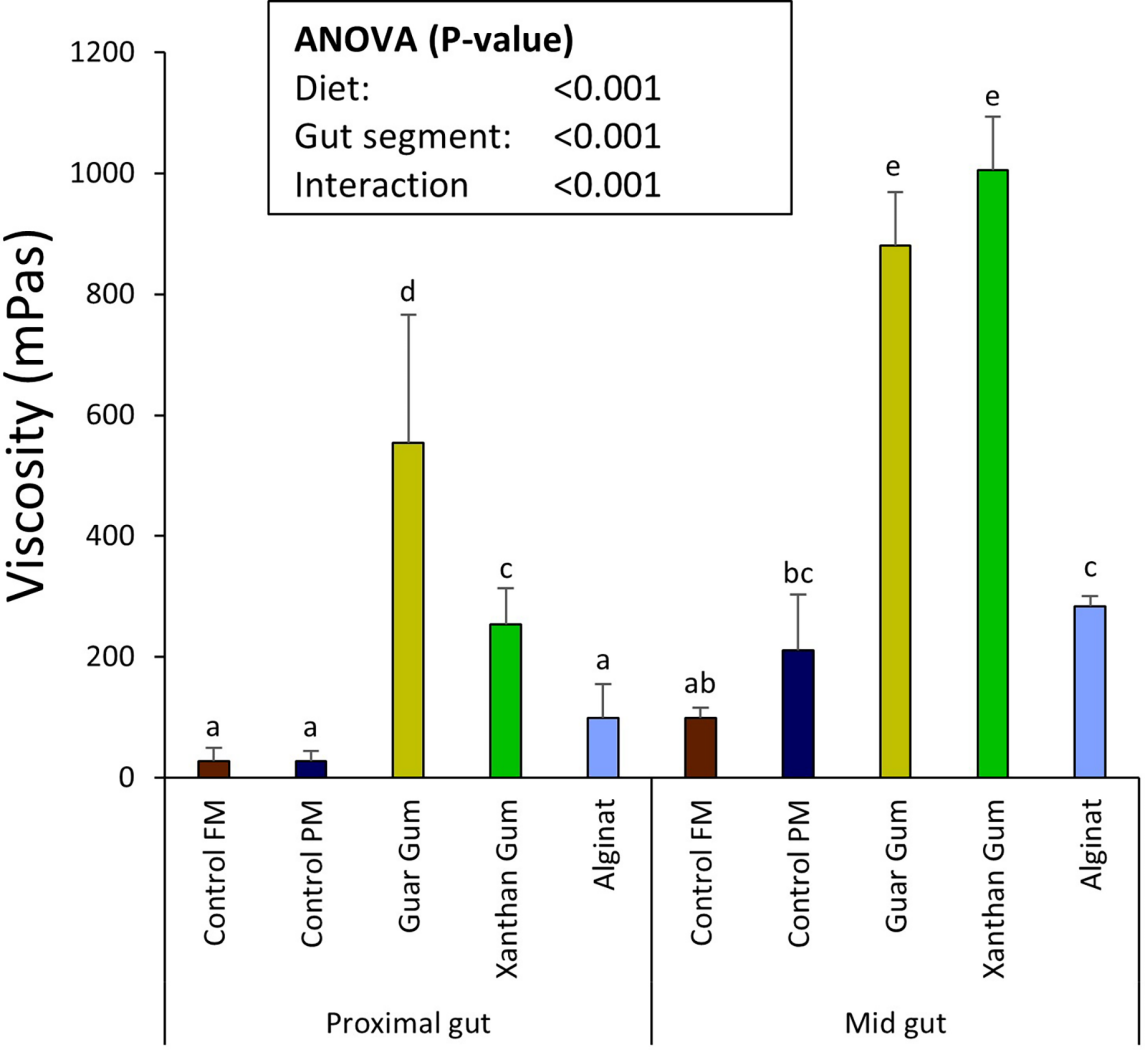
Average viscosity at 20 °C in pooled content from proximal and mid gut from dissected Atlantic salmon (10 per tank) fed either control feeds with PM, FM or 2% inclusion of guar gum, xanthan gum or alginate in triplicate tanks for a period of 14 days. Mean ± standard deviation, *n* = 3. Statistics by two-way ANOVA; followed by Tukey *post hoc* test. Data were log-transformed prior to analysis due to variance heterogeneity (Levene’s test). Superscripts showing different letters are significantly different (*P* < 0.05).

Proximal gut viscosity was highest in salmon fed guar gum, intermediate in those fed xanthan gum, and lowest in all remaining feeds. Mid gut viscosity was similarly high in salmon fed guar gum and xanthan gum, intermediate in the alginate group, and significantly lower in both control feeds (ANOVA *P* < 0.05; Fig. 4).

Dry matter content tended to be higher across the gut of salmon fed the FM control feed (Table 4). Dry matter levels did not differ significantly among feeds in the proximal gut, but both control feeds had higher dry matter in the mid gut. In this section, all feeds with binders produced reduced dry matter content. Distal gut dry matter was significantly lower in salmon fed feeds xanthan gum and alginate feeds compared to the proximal gut (ANOVA; *P* < 0.05), with a similar but non-significant trend in salmon fed guar gum (ANOVA; *P* = 0.144). Distal gut dry matter was significantly lower in the guar gum and xanthan gum groups (14.5%) compared to the FM control (19.1%), which was also significantly higher than in the PM control group (16.3%; Tukey *post hoc*; *P* < 0.05).

**Table 4:** Content of dry matter (DM; %), ash (%) and yttrium (mg/kg) in content from different GI segments of dissected Atlantic salmon (10 per tank) fed either control feeds with PM, FM or 2% inclusion of guar gum, xanthan gum or alginate in triplicate tanks for a period of 14 days.

DM	Proximal gut	Mid gut	Distal gut	ANOVA (*P*-value)
Control FM	18.1 ± 0.6^a^	19.8 ± 1.1^c^	19.1 ± 0.4^c^	0.089
Control PM	16.2 ± 1.7^a^	18.0 ± 0.8^bc^	16.3 ± 0.2^b^	0.169
Guar gum	17.3 ± 2.2^a^	15.8 ± 0.8^a^	14.6 ± 0.9^a^	0.144
Xanthan gum	17.5 ± 0.7^a^	17.1 ± 0.5^ab^	14.4 ± 0.2^a^	0.001
Alginate	17.3 ± 0.4^a^	16.9 ± 0.3^ab^	15.3 ± 0.5^ab^	0.003
**Ash**	**Proximal gut **	**Mid gut **	**Distal gut **	
Control FM	2.9 ± 0.2^b^	4.6 ± 0.3^c^	N.A.	0.001
Control PM	2.2 ± 0.3^a^	3.1 ± 0.1^b^	N.A.	0.009
Guar gum	2.2 ± 0.1^a^	2.4 ± 0.1^a^	N.A.	0.009
Xanthan gum	2.3 ± 0.0^a^	2.9 ± 0.1^b^	N.A.	<0.001
Alginate	2.2 ± 0.1^a^	2.9 ± 0.1^b^	N.A.	0.001
**Yttrium**	**Proximal gut **	**Mid gut **	**Distal gut **	
Control FM	106 ± 14^a^	225 ± 11^b^	370 ± 7^c^	<0.001
Control PM	121 ± 21^a^	239 ± 8^b^	323 ± 2^b^	<0.001
Guar gum	125 ± 12^a^	185 ± 15^a^	220 ± 3^a^	0.002
Xanthan gum	133 ± 12^a^	235 ± 8^b^	286 ± 6^b^	<0.001
Alginate	120 ± 3^a^	216 ± 4^b^	282 ± 1^b^	<0.001

**Note:**

Mean ± standard deviation, *n* = 3. N.A. = not analysed. Statistics by ANOVA, significant differences (*P* < 0.05) between gut segment in left column and superscript letters showing differences between dietary groups within the same gut segment analysed by Tukey *post hoc* test. Data were log-transformed prior to analysis due to variance heterogeneity (Levene’s test).

Ash content increased from the proximal gut to the mid gut (ANOVA; *P* < 0.05) and was significantly higher in salmon fed FM control feed than those fed other feeds. The lowest ash content was observed in salmon fed guar gum (Tukey *post hoc*; *P* < 0.05). Yttrium concentration also increased along the GI tract, and significantly lower concentration in salmon fed guar gum indicated reduced digestibility in both the mid and distal gut compared to the other feeds (Table 4).

Visual assessment of distal gut content revealed generally solid to semi-soft digesta with fragile structures, and substantial individual differences. No significant differences in faecal score were detected (Table S3), but visual observations revealed dietary effects (Table S4). Salmon fed guar gum produced clearly mucus-coated faecal pellets, while salmon fed the FM control and xanthan gum fed produced more segmented faecal mass, and salmon fed PM control and alginate created more homogenous digesta lacking distinct pellets (Table 5). The mucus coating differed between FM control and guar gum feeds.

**Table 5:** Picture of dissected faecal samples from distal gut, selected samples per feeding group.

Control FM	Control PM	Guar gum	Xanthan gum	Alginate
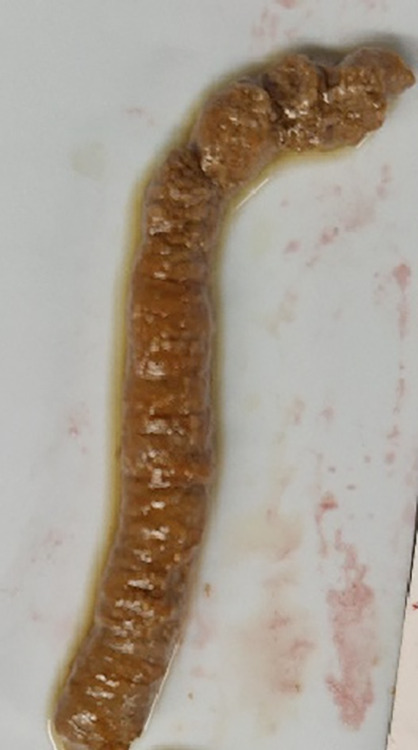	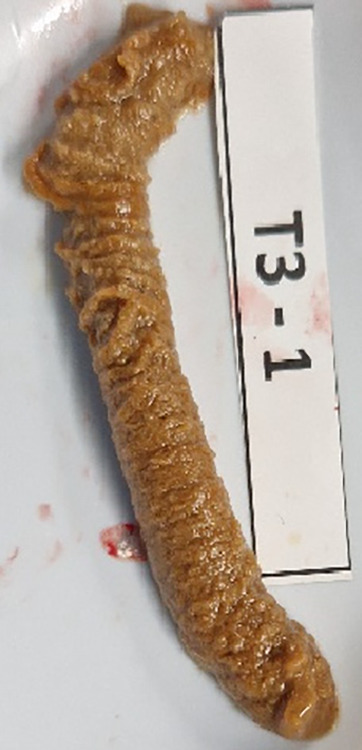	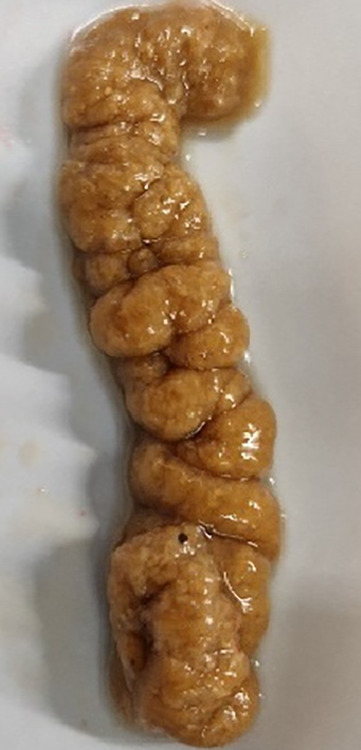	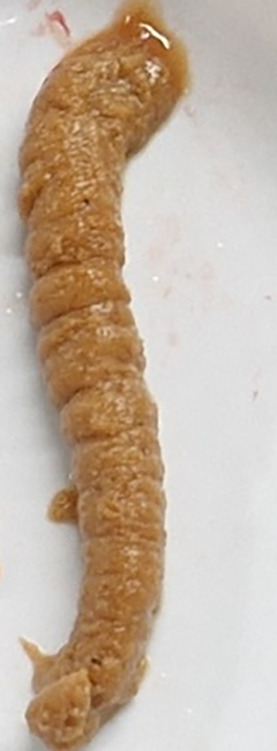	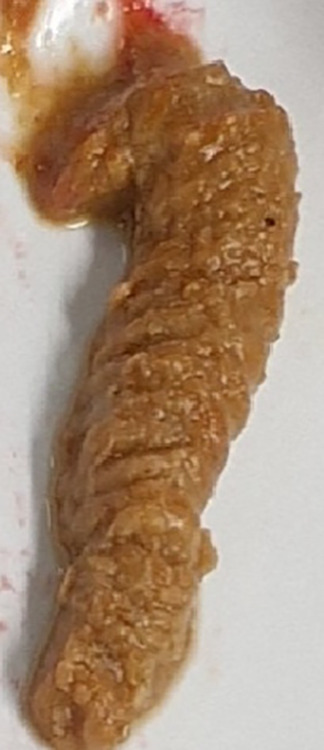

### Digestibility of nutrients as a response of feeds and collection method

**Figure 5:**
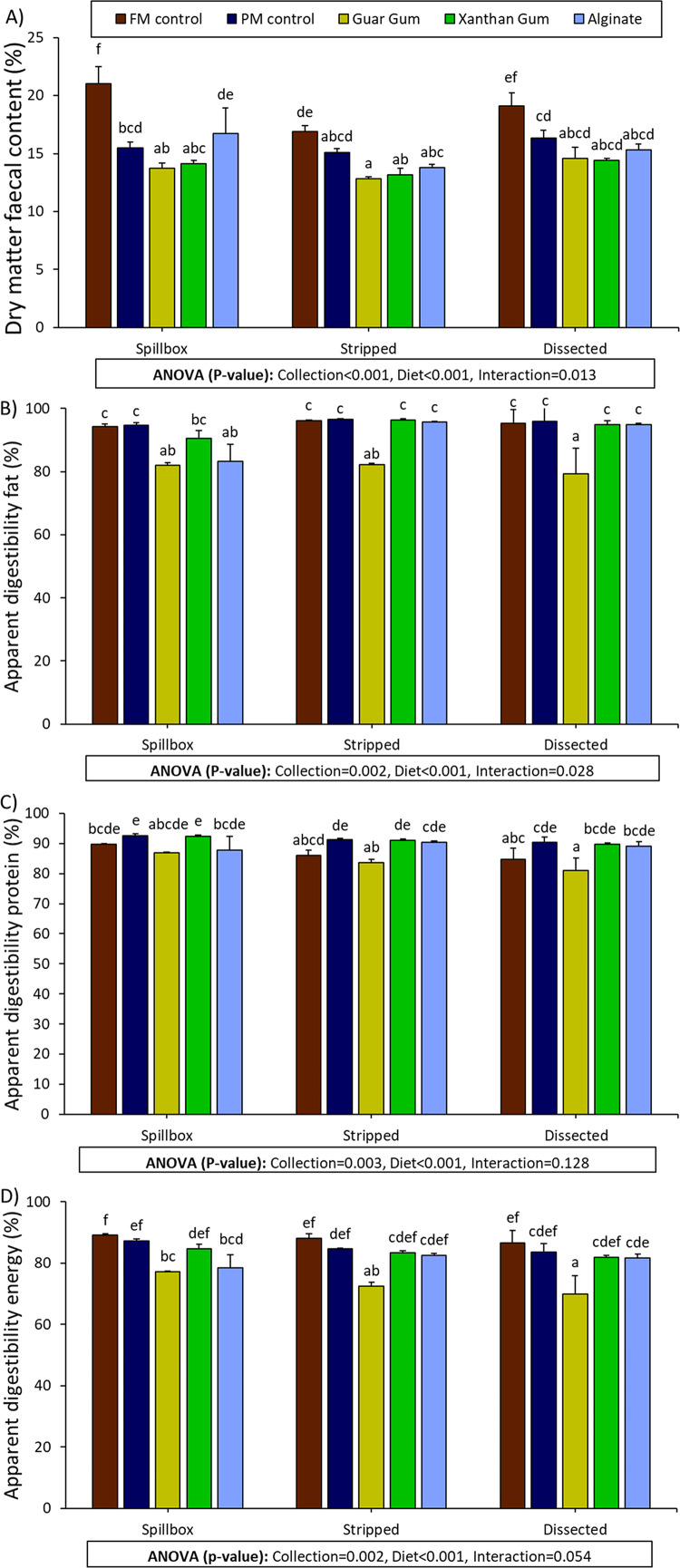
Faecal dry matter (A) and apparent digestibility of fat (B), protein (C) and energy (D) from Spillbox content, distal gut content collected by dissection (10 fish per tank) or faeces collected by stripping (20 fish per tank). Mean ± standard deviation, *n* = 3. Statistics by two-way ANOVA; followed by Tukey *post hoc* test. Data for DM%, ADC fat and protein of dissected content and ADC fat of stripped content were log-transformed prior to analysis due to variance heterogeneity (Levene’s test). Superscripts showing different letters are significant different (*P* < 0.05).

Faeces obtained by stripping had consistently lower dry matter content than faeces collected in the Spillbox and by dissection (ANOVA; *P* < 0.05). Across all collection methods, faeces from the FM control feed had significantly higher dry matter content than faeces from salmon fed the other feeds (ANOVA; *P* < 0.05; Fig. 5A). Apparent digestibility for protein, fat and energy differ among collection methods and feeds (ANOVA; *P* < 0.05; Figs. 5B, 5D). Salmon fed 2% guar gum had significantly lower digestibility than all other groups. Furthermore, this group showed also the largest discrepancy among collection methods, with higher digestibility estimates from the Spillbox samples, intermediate values from the stripped samples and the lowest from dissected contents (Fig. 5).

### Faecal collection efficiency of feeds with different inclusion of FM and binders

**Figure 6:**
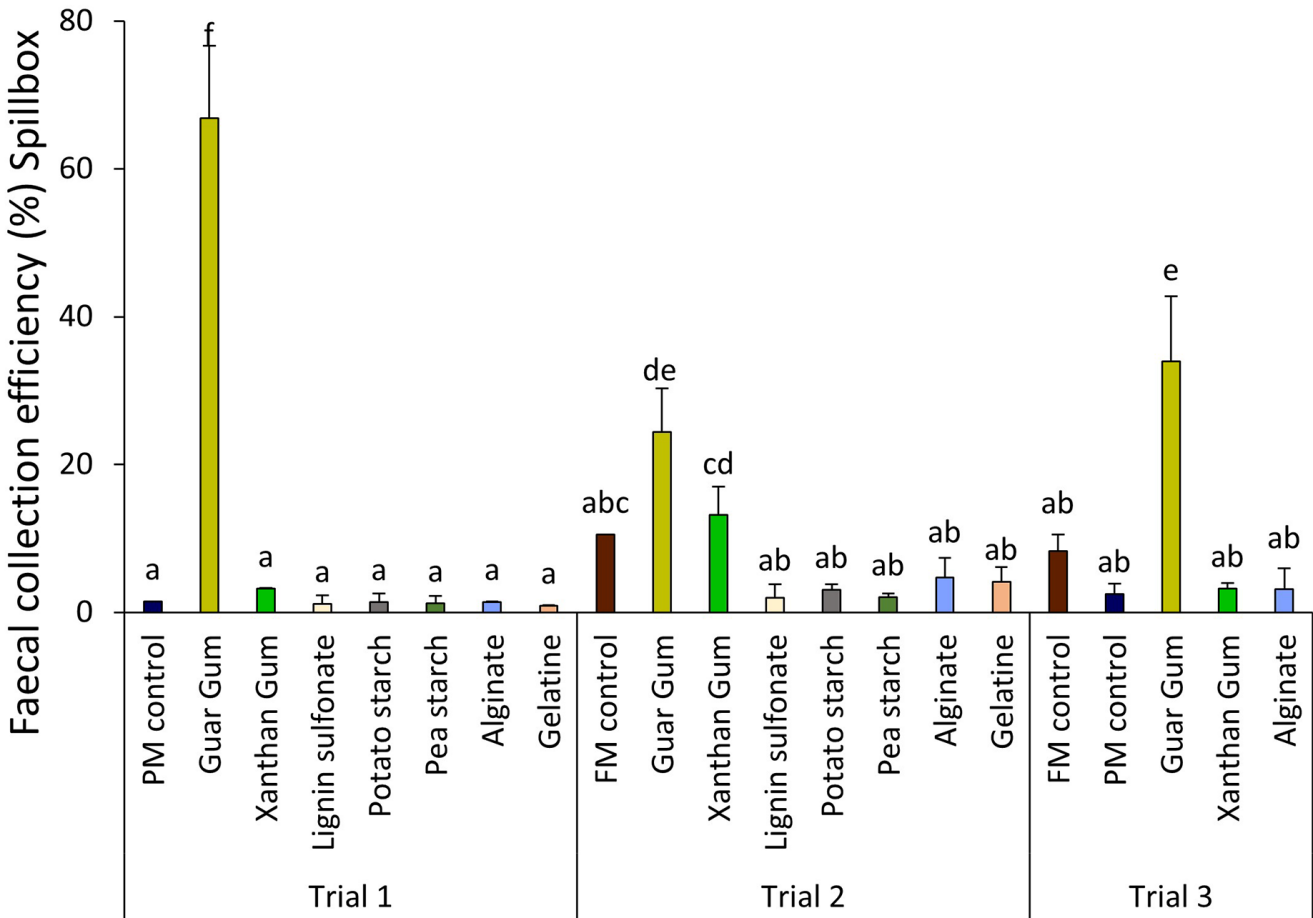
Faecal collection efficiency of dry matter in Spillbox. Mean ± standard deviation. Statistics by two-way ANOVA; followed by Tukey *post hoc* test. Data were log-transformed prior to analysis due to variance heterogeneity (Levene’s test). Superscripts showing different letters are significant different (*P* < 0.05).

Faecal collection efficiency was calculated as the percentage of total dry matter collected in the Spillbox relative to the total estimated faecal matter produced per tank. Because digestibility measurements were not available for Trial 1 and 2, a dry matter digestibility of 70% was applied for efficiency estimates. The guar gum feed produced faecal collection efficiency from 20–73%, with mean values at 67% and 34% at 2% inclusion in Trial 1 and 3, respectively, and 24% at 5% inclusion in Trial 2. In contrast, xanthan gum at 5% inclusion significantly increased collection efficiency (11%) compared to 2% inclusion (3%). The other binders showed only minor changes in efficiency with increasing dietary inclusion, and overall collection efficiencies remained below 5%. The FM control feed had collection efficiency comparable to the 5% xanthan gum inclusion, with values of 11% in Trial 2 and 8% in Trial 3 (Fig. 6).

**Figure 7:**
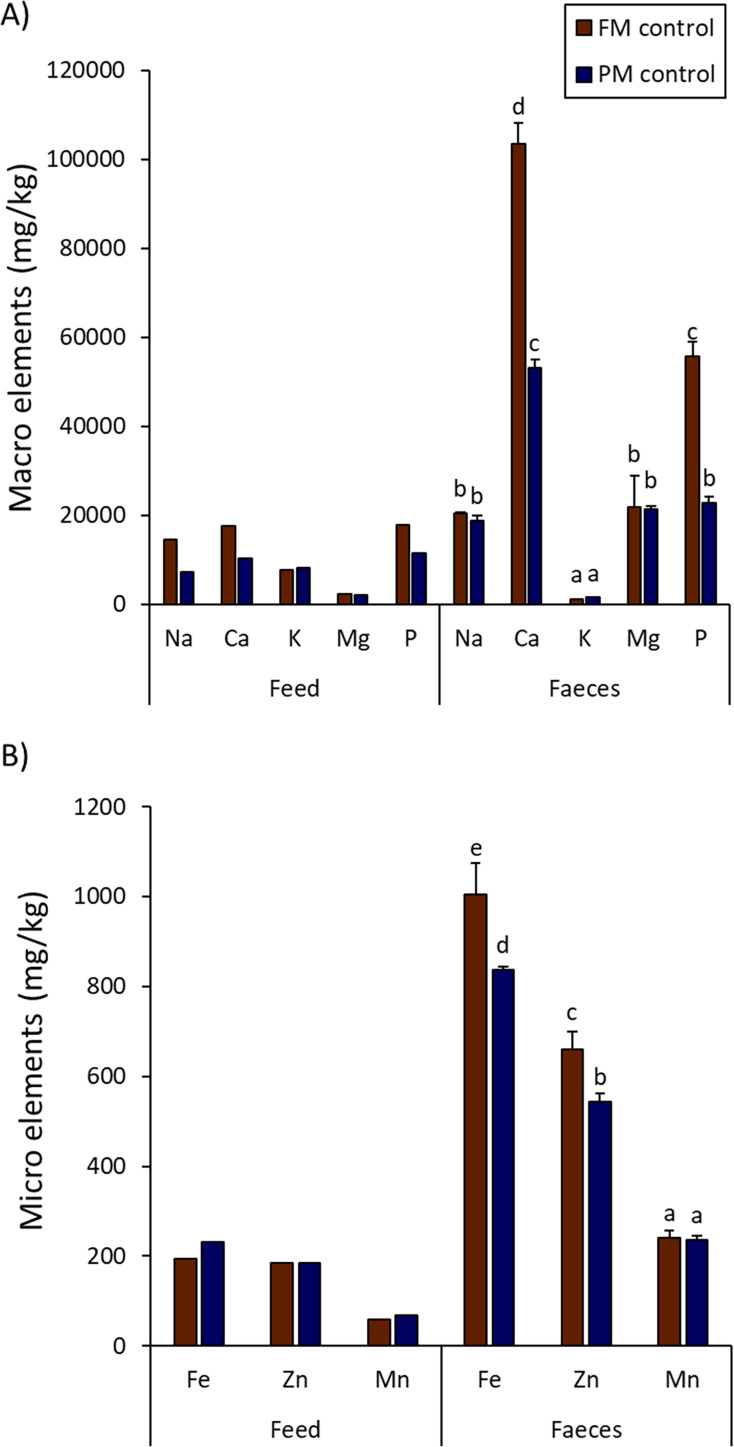
Micro and macro mineral composition in feed and faeces collected by stripping (20 fish per tank). Mean ± standard deviation, *n* = 3. Statistics in mineral composition of faecal samples by two-way ANOVA mean effect of mineral elements (*P* < 0.05) and feed (*P* < 0.05); followed by Tukey *post hoc* test. Data were log-transformed prior to analysis due to variance heterogeneity (Levene’s test). Superscripts showing different letters are significant different (*P* < 0.05).

Mineral composition differences between the FM and PM control feeds were reflected in faecal mineral profiles, with salmon fed FM feed showing higher concentrations of iron, zinc, calcium and phosphorous (Fig. 7). Binder inclusion in the PM feed was assumed not to have a substantial impact on mineral composition, as similar ash content was observed in these feeds and in the corresponding faeces samples.

## Discussion

The feeds containing the binders guar gum, xanthan gum, lignin sulfonate, potato starch, pea starch, alginate and gelatine all exhibited high water stability, which is advantageous for the collection of uneaten feed in the Spillbox system. However, binder inclusion affected faecal properties differently in the salmon. Guar gum, a well-established binder in aquaculture feeds, particularly for RAS (Storebakken, 1985; Brinker, 2007), most effectively improved faeces collection compared with the other binders tested. High inclusion of xanthan gum (5%) and the FM control feed also appeared to influence faecal characteristics and increased the ability to retain faeces in the Spillbox. Viscosity profiles (ViscoQuick) showed partial correspondence with faecal properties for guar gum and xanthan gum, both yielding feeds with higher viscosity, but these measurements did not explain the improved faecal characteristics observed in salmon fed the FM control feed, which had the lowest viscosity values.

Improved mass-balance control of nutrients and waste is essential for increased resource efficiency and establishing more circular food systems in aquaculture (Timmons, Guerdat & Vinci, 2018). Current limitations in land-based farms are largely related to inefficient waste-collection technologies. Systems such as the custom-designed Spillbox, which removes feed and faeces directly at the tank outlet, represent a potential solution for enhancing recovery and utilization of waste resources. Future farming systems should also incentivize farmers to improve waste management by minimizing overfeeding and using highly digestible feeds (Amirkolaie, 2011), as well as promoting the development of feeds formulations that enhance faecal collection efficiency (Prakash et al., 2023).

Binders such as starch, cellulose and guar gum have long been known to influence faecal properties in fish (Storebakken, 1985; Dias et al., 1998). The binders selected for this study also included compounds commonly used in the food industry, such as gelatine and alginate (Abdallah et al., 2018). Inclusion levels were selected to generate clear contrasts in faecal collection performance. Although a previous study showed that as little as 0.3% guar gum improved faecal stability and water quality in RAS for rainbow trout (*Oncorhynchus mykiss*) (Brinker, 2007), sufficient inclusion levels for the other binders were not known. In the present study, 2% inclusion of binders other than guar gum did not provide adequate faecal stability for Spillbox collection, whereas increasing inclusion to 5% improved collection efficiency for most binders, particularly xanthan gum.

In contrast, guar gum showed higher collection efficiency at lower inclusion level. The greater collection efficiency at 2% guar gum (Trial 1) compared to 5% (Trial 2) may reflect reduced gastrointestinal passage rate associated with lower feed intake. Slower passage enhances nutrient and water absorption, which likely contributed to the drier faeces and higher recovery observed in Trial 1. This interpretation aligns with Bogevik et al. (2021), who reported that faster pellet disintegration increased feed intake and GI transit rate and tended to reduce nutrient digestibility. Across trials, tanks receiving guar gum consistently showed higher Spillbox collection efficiency than all dietary treatments, and these salmon also produced larger sample volumes during distal gut dissection and stripping.

Gastrointestinal filling is known to influence feeding regime. Studies in gilthead seabream (*Sparus aurata*) and Senegalese sole (*Solea senegalensis*) have shown that a single large daily meal increased gut filling and reduced digestibility relative to more frequent feeding (Gilannejad et al., 2019). Feed characteristics such as particle size, nutrient composition, and physical quality also affect gut filling, passage rate and digestibility (Sveier, Wathne & Lied, 1999; Dias et al., 2010; Aas et al., 2021). Because feeding regime and pellet physical quality were similar among feeds, differences in GI response in the present study were likely driven by the inherited chemical structures provided by inclusion of the binders.

Dissection of GI content at the end of Trial 3 revealed dietary differences in the total amount and distribution of digesta. Salmon fed the alginate feed appeared to have lower overall GI content but proportionally higher content in the stomach and distal gut. Total feed intake was although high in this group, which may indicate that this pattern was due to a temporary reduction in feeding shortly before sampling, resulting in less mid-intestinal content at dissection.

Viscosity and dry matter normally increases from the proximal to the distal gut (Bogevik et al., 2021), as observed for FM control, while feeds with binders produced lower dry matter content throughout the gut. The guar gum feed produced the highest viscosity in proximal gut content, with a further increase together with the xanthan gum in the mid gut. Increased intestinal viscosity has been associated with reduced nutrient digestibility, as demonstrated for non-starch polysaccharide from soybean in salmon feeds (Refstie et al., 1999) and for guar gum in Nile tilapia (*Oreochromis niloticus*; Amirkolaie et al., 2005). Elevated viscosity likely impairs enzyme activity, hydrolysis and nutrient absorption (Smits & Annison, 1996). Therefore, these binders should be used at low inclusion levels to avoid negative effects on feed intake, digestion and nutrient utilisation.

Nutrient digestibility in this study was evaluated using three faeces collection methods: Spillbox collection, stripping, and distal gut dissection. Spillbox samples had higher dry matter content, likely due to loss of water-soluble components during transport in water and evaporation of the collected samples exposed to air, whereas stripped samples may have contained additional mucus and water from the skin. These differences influence digestibility estimates, with Spillbox samples potentially overestimating digestibility and stripped samples potentially underestimating it, which also is true for dissected samples if nutrient absorption continues in the distal gut. Within dietary groups, dissected samples consistently produced lower protein and energy digestibility than Spillbox samples. Nevertheless, dietary effects were more pronounced than methodological effects. Across all methods, salmon fed guar gum exhibited significantly lower digestibility of dry matter, fat, protein and energy than salmon fed other feeds. Although each method has limitations, stripping remains a valuable method for accurate nutrient digestibility assessment, particularly for feeds with water-soluble protein fractions. The Spillbox system provides a practical, non-invasive means of monitoring digestion under conditions that more closely resemble farm production, and a scalable solution for monitoring waste output in commercial farms.

Although guar gum produced the most consistent improvement in faecal collection efficiency, the effects of xanthan gum and feed ingredients such as FM require further investigation. The FM control feed resulted in high nutrient digestibility yet also produced one of the highest amounts of recoverable faecal waste. Compared with PM-based feeds, the FM feed contained higher mineral levels and lower carbohydrate levels. Reduced distal gut dry matter and collection efficiency in PM feeds may be related to indigestible non-starch polysaccharide, as seen in other species (Brinker & Friedrich, 2012; Prakash et al., 2025). Minerals generally have low digestibility (Lall & Kaushik, 2021) and may influence water reabsorption and faecal dry matter content. However, little is known about the effects of dietary mineral content on digestive function connected to faecal characteristics in Atlantic salmon. In postsmolt salmon, increased gut mineral concentration (Ca, Na, K, Cl) during salinity adaptation has been associated with reduce proximal-gut dry matter and protein digestibility (Ciavoni et al., 2024). Dietary minerals may therefore influence water reabsorption, distal dry matter content, viscosity, gelling behaviour, water-binding capacity, and overall faecal properties. Studies in rainbow trout have shown that higher dietary ash content, resulting from increased fish meal or diamol inclusion, increased faecal density and sinking velocity (Prakash et al., 2023, 2024), although this did not improved faecal removal efficiency as observed here. The authors attributed the increased density to higher inorganic minerals content; however, our results suggest that improved faecal recovery may instead reflect enhanced water reabsorption and formation of more cohesive faecal pellets, potentially mediated by mucus encapsulation in the distal gut.

## Conclusions

This study demonstrated that guar gum and xanthan gum can be used to modify intestinal viscosity and dry matter content, thereby improving faecal removal efficiency under controlled conditions. High fish meal inclusion also enhanced faecal properties, likely due to increased dry matter and a higher minerals-to-carbohydrate ratio. The Spillbox system proved effective for capturing faeces and uneaten feed at the tank outlet and may better represent future improvements in localized waste capture within aquaculture facilities. In contrast, centralized collection systems in commercial land-based farms often involve longer transport distances, increasing the risk of faecal disintegration and reduced collection efficiency. These findings highlight the importance of both feed formulation and system design in improving solid waste management. Further research should explore the combined effects of dietary minerals with carbohydrate-based binders, such as guar gum, to enhance faecal collection efficiency in Atlantic salmon under commercial conditions.

## Supplemental Information

10.7717/peerj.21291/supp-1 Supplemental Information 1 Supplementary Tables.

10.7717/peerj.21291/supp-2 Supplemental Information 2 Raw Data.

10.7717/peerj.21291/supp-3 Supplemental Information 3 ARRIVE checklist.

## Additional Information and Declarations

### Data Availability

The following information was supplied regarding data availability:

The raw data is available in the Supplemental File.

## References

[ref-1] Aas TS, Ytrestøyl T, Berge GM (2016). Dry matter content in sludge from land-based production of Atlantic salmon (Norwegian). https://hdl.handle.net/11250/2402326.

[ref-2] Aas TS, Ytrestøyl T, Sixten HJ, Hillestad M, Sveier H, Åsgård T (2021). Physical feed properties affect gastrointestinal passage rate in Atlantic salmon, Salmo salar. Aquaculture Nutrition.

[ref-3] Aas TS, Åsgård T (2019). Material flow of nutrients and energy from feed in a land-based hatchery (Norwegian). https://hdl.handle.net/11250/2587104.

[ref-4] Aas TS, Åsgård T, Ytrestøyl T (2022). Utilization of feed resources in the production of Atlantic salmon (Salmo salar) in Norway: an update for 2020. Aquaculture Reports.

[ref-5] Abdallah MR, Mohamed MA, Mohamed H, Emara MT (2018). Application of alginate and gelatin-based edible coating materials as alternatives to traditional coating for improving the quality of pastirma. Food Science and Biotechnology.

[ref-6] Ahmad R, Oterhals Å, Xue Y, Skodvin T, Samuelsen TA (2019). Impact of fish protein concentrate on apparent viscosity and physical properties of soy protein concentrate subjected to thermomechanical treatment. Journal of Food Engineering.

[ref-7] Amirkolaie AK (2011). Reduction in the environmental impact of waste discharged by fish farms through feed and feeding. Reviews in Aquaculture.

[ref-8] Amirkolaie AK, Leenhouwers JI, Verreth JAJ, Schrama JW (2005). Type of dietary fibre (soluble versus insoluble) influences digestion, faeces characteristics and faecal waste production in Nile tilapia (Oreochromis niloticus L.). Aquaculture Research.

[ref-9] Amirkolaie AK, Verreth JAJ, Schrama JW (2006). Effect of gelatinization degree and inclusion level of dietary starch on the characteristics of digesta and faeces in Nile tilapia (Oreochromis niloticus (L.)). Aquaculture.

[ref-10] Austreng E (1978). Digestibility determination in fish using chromic oxide marking and analysis of contents from different segments of the gastrointestinal tract. Aquaculture.

[ref-11] Baeverfjord G, Krogdahl A (1996). Development and regression of soybean meal induced enteritis in Atlantic salmon, Salmo salar L., distal intestine: a comparison with the intestines of fasted fish. Journal of Fish Diseases.

[ref-12] Baeverfjord G, Refstie S, Krogedal P, Åsgård T (2006). Low feed pellet water stability and fluctuating water salinity cause separation and accumulation of dietary oil in the stomach of rainbow trout (Oncorhynchus mykiss). Aquaculture.

[ref-13] Bogevik AS, Samuelsen TA, Aspevik T, Romarheim OH, Aas TS, Kalananthan T, Rønnestad I (2021). Disintegration stability of extruded fish feed affects gastric functions in Atlantic salmon (*Salmo salar*). Aquaculture.

[ref-14] Brinker A (2007). Guar gum in rainbow trout (*Oncorhynchus mykiss*) feed: the influence of quality and dose on stabilisation of faecal solids. Aquaculture.

[ref-15] Brinker A (2009). Improving the mechanical characteristics of faecal waste in rainbow trout: the influence of fish size and treatment with a non-starch polysaccharide (guar gum). Aquaculture Nutrition.

[ref-16] Brinker A, Friedrich C (2012). Fish meal replacement by plant protein substitution and guar gum addition in trout feed. Part II: effects on faeces stability and rheology. Biorheology.

[ref-17] Ciavoni E, Schrama JW, Radhakrishnan G, Sæle Ø, Prabhu Philip AJ (2024). Salinity induced changes in the progression of water, ion and nutrient fluxes along the gastrointestinal tract of Atlantic salmon smolt (*Salmo salar*). Aquaculture.

[ref-18] Dias J, Huelvan C, Dinis MT, Métailler R (1998). Influence of dietary bulk agents (silica, cellulose and a natural zeolite) on protein digestibility, growth, feed intake and feed transit time in European seabass (*Dicentrarchus labrax*) juveniles. Aquatic Living Resources.

[ref-19] Dias J, Yúfera M, Valente LMP, Rema P (2010). Feed transit and apparent protein, phosphorus and energy digestibility of practical feed ingredients by Senegalese sole (*Solea senegalensis*). Aquaculture.

[ref-20] European Commission (2009). Commission Regulation (EC) No 152/2009 of 27 January 2009 laying down the methods of sampling and analysis for the official control of feed. Official Journal of the European Union.

[ref-21] Gilannejad N, Silva T, Martínez-Rodríguez G, Yúfera M (2019). Effect of feeding time and frequency on gut transit and feed digestibility in two fish species with different feeding behaviours, gilthead seabream and Senegalese sole. Aquaculture.

[ref-22] Lall SP, Kaushik SJ (2021). Nutrition and metabolism of minerals in fish. Animals.

[ref-23] Meriac A, Eding EH, Schrama JW, Kamstra A, Verreth JAJ (2014). Dietary carbohydrate composition can change waste production and biofilter load in recirculating aquaculture systems. Aquaculture.

[ref-24] Pearce CM, Daggett TL, Robinson SMC (2002). Effect of binder type and concentration on prepared feed stability and gonad yield and quality of the green sea urchin, *Strongylocentrotus droebachiensis*. Aquaculture.

[ref-25] Prakash S, Maas RM, Bergersen A, Kals J, Kokou F, Schrama JW, Prabhu Philip AJ (2025). Dietary starch, non-starch polysaccharides and their interactions affect nutrient digestibility, faecal waste production and characteristics differentially in three salmonids: rainbow trout, Atlantic salmon and Arctic charr. Aquaculture.

[ref-26] Prakash S, Maas RM, Fransen P-MMM, Kokou F, Schrama JW, Philip AJP (2023). Effect of feed ingredients on nutrient digestibility, waste production and physical characteristics of rainbow trout (*Oncorhynchus mykiss*) faeces. Aquaculture.

[ref-27] Prakash S, Maas RM, Horstmann P, Elbers JJ, Kokou F, Schrama JW, Philip AJP (2024). Effect of dietary starch, amylase and ash on nutrient digestibility, faecal waste production and faecal characteristics of rainbow trout, (*Oncorhynchus mykiss*). Aquaculture.

[ref-28] Refstie S, Svihus B, Shearer KD, Storebakken T (1999). Nutrient digestibility in Atlantic salmon and broiler chickens related to viscosity and non-starch polysaccharide content in different soyabean products. Animal Feed Science and Technology.

[ref-29] Samuelsen TA, Mjøs SA, Oterhals Å (2013). Impact of variability in fishmeal physicochemical properties on the extrusion process, starch gelatinization and pellet durability and hardness. Animal Feed Science and Technology.

[ref-30] Samuelsen TA, Mjøs SA, Oterhals Å (2014). Influence of type of raw material on fishmeal physicochemical properties, the extrusion process, starch gelatinization and physical quality of fish feed. Aquaculture Nutrition.

[ref-31] Samuelsen TA, Oterhals Å (2016). Water-soluble protein level in fishmeal affects extrusion behaviour, phase transitions and physical quality of feed. Aquaculture Nutrition.

[ref-32] Smits CHM, Annison G (1996). Non-starch plant polysaccharides in broiler nutrition—towards a physiologically valid approach to their determination. World’s Poultry Science Journal.

[ref-35] Sørensen M (2012). A review of the effects of ingredient composition and processing conditions on the physical qualities of extruded high-energy fish feed as measured by prevailing methods. Aquaculture Nutrition.

[ref-33] Storebakken T (1985). Binders in fish feeds: I. Effect of alginate and guar gum on growth, digestibility, feed intake and passage through the gastrointestinal tract of rainbow trout. Aquaculture.

[ref-34] Sveier H, Wathne E, Lied E (1999). Growth, feed and nutrient utilisation and gastrointestinal evacuation time in Atlantic salmon (*Salmo salar* L.): the effect of dietary fish meal particle size and protein concentration. Aquaculture.

[ref-36] Timmons M, Guerdat T, Vinci B (2018). Recirculating aquaculture.

[ref-37] Welker TL, Overturf K, Barrows F (2020). Development and evaluation of a volumetric quantification method for fecal particle size classification in rainbow trout fed different diets. North American Journal of Aquaculture.

[ref-38] Zhao Y, Qiu J, Xu J, Gao X, Fu X (2017). Effects of crosslinking modes on the film forming properties of kelp mulching films. Algal Research.

