# Supplementary material for: Feed binders to improve the quality of Atlantic salmon (Salmo salar) feed and faeces
Source: PeerJ. 2026 Jun 22;14:e21291. doi: 10.7717/peerj.21291 (PMC13296804; doi:10.7717/peerj.21291)
Supplement: Supplemental Information 1 [file peerj-14-21291-s001.docx]

**Supplementary Tables and Figures**

**Table 1.** ViscoQuick cold, peak, hold and final viscosity for the plant meal (PM) and fishmeal (FM) feed mixes and feed mixes containing three selected binders at 2% inclusion level.

|  | Control PM | Control FM | Guar Gum | Xanthan Gum | Alginate | *P*-value |
| --- | --- | --- | --- | --- | --- | --- |
| Cold viscosity (cP) | 33.6 ± 6.8^a^ | 26.3 ± 1.9^a^ | 572.3 ± 21.4^d^ | 409.3 ± 1.9^c^ | 145.9 ± 2.4^b^ | <0.0001 |
| Peak viscosity (cP) | 360.8 ± 17.8^b^ | 134.7 ± 1.5^a^ | 1071.9 ± 37.5^d^ | 751.1 ± 2.2^c^ | 366.1 ± 7.8^b^ | <0.0001 |
| Hold viscosity (cP) | 249.8 ± 0.2^b^ | 105.6 ± 1.3^a^ | 747.9 ± 25.2^e^ | 497.1 ± 5.9^d^ | 302.5 ± 1.1^c^ | <0.0001 |
| Final viscosity (cP) | 493.9 ± 2.3^b^ | 234.5 ± 3.2^a^ | 2333.2 ± 13.6^e^ | 1389.4 ± 28.6^d^ | 912.5 ± 28.3^c^ | <0.0001 |

Mean ± standard deviation; n = 2. Statistics by one-way ANOVA for viscosity effects at P < 0.05, followed by Tukey post hoc test. Superscripts showing different letters are significantly different.

**Table 2.** Pellet hardness and water stability index for all feeds.

| Feeds | Hardness | Water stability index |
| --- | --- | --- |
| PM control | 59.9±7.2^fg^ | 86.5±0.1^c^ |
| FM control | 27.5±2.8^a^ | 83.0±0.2^b^ |
| 2% Guar Gum | 42.3±3.6^b^ | 88.0±0.3^defg^ |
| 2% Xanthan | 49.9±7.5^bcde^ | 87.3±0.2^cdef^ |
| 2% Lignin | 46.5±3.8^bcd^ | 88.4±0.1^fgh^ |
| 2% Potato starch | 56.1±6.7^ef^ | 88.4±0.2^efgh^ |
| 2% Pea starch | 50.6±3.6^de^ | 88.8±0.1^gh^ |
| 2% Alginate | 47.8±3.9^bcd^ | 87.9±0.2^cdefg^ |
| 2% Gelatine | 76.9±7.6^h^ | 88.1±0.4^defg^ |
| 5% Guar Gum | 42.8±5.2^bc^ | 88.5±0.1^fgh^ |
| 5% Xanthan | 50.1±7.3^cde^ | 86.9±0.2^cde^ |
| 5% Lignin | 59.4±6.0^fg^ | 87.2±0.2^cdef^ |
| 5% Potato starch | 65.8±5.2^g^ | 86.8±1.3^cd^ |
| 5% Pea starch | 62.3±8.7^fg^ | 87.6±0.1^cdefg^ |
| 5% Alginate | 73.7±9.0^h^ | 79.7±0.4^a^ |
| 5% Gelatine | 112.9±14.9^i^ | 89.8±0.3^h^ |
| *p*-value | <0.0001 | 0.0073 |

Mean ± standard deviation; n = 20 for hardness and n = 3 for water stability index. Statistics by one-way ANOVA for the effects at P < 0.05, followed by Tukey post hoc test. Superscripts showing different letters are significantly different for each of the analyses.

**Table 3.** Biometric data and gastrointestinal filling in sampled Atlantic salmon males and females fed feeds with different binders at termination of Trial 3.

| **Feeds** | **Control FM** | | **Control PM** | | **Guar gum 2%** | | **Xanthan gum 2%** | | **Alginate 2%** | |  | **ANOVA** | |  |
| --- | --- | --- | --- | --- | --- | --- | --- | --- | --- | --- | --- | --- | --- | --- |
| **Sex** | **Females** | **Males** | **Females** | **Males** | **Females** | **Males** | **Females** | **Males** | **Females** | **Males** |  | **Sex** | **Feed** | **Sex x Feed** |
| Number of fish | 13 | 17 | 9 | 21 | 13 | 17 | 11 | 19 | 10 | 20 |  |  | | |
| Body weight (BW, g) | 594±87^a^ | 613±84^a^ | 543±117^a^ | 595±115^a^ | 561±89^a^ | 539±97^a^ | 570±89^a^ | 598±101^a^ | 613±57^a^ | 611±107^a^ |  | 0.382 | 0.117 | 0.688 |
| Fork length (cm) | 35.2±1.4^a^ | 36.0±1.1^a^ | 34.7±2.3^a^ | 35.8±1.6^a^ | 35.2±1.5^a^ | 34.7±1.9^a^ | 35.4±1.5^a^ | 35.5±1.9^a^ | 36.0±1.0^a^ | 36.0±2.0^a^ |  | 0.295 | 0.206 | 0.451 |
| K-factor | 1.4±0.1^a^ | 1.3±0.1^a^ | 1.3±0.1^a^ | 1.3±0.1^a^ | 1.3±0.1^a^ | 1.3±0.1^a^ | 1.3±0.1^a^ | 1.3±0.1^a^ | 1.3±0.1^a^ | 1.3±0.1^a^ |  | 0.248 | 0.785 | 0.463 |
| Total GI content (% of BW) | 9.6±2.8^a^ | 9.4±2.8^a^ | 10.4±4.9^a^ | 10.2±4.0^a^ | 11.3±3.6^a^ | 11.0±4.0^a^ | 10.7±2.8^a^ | 10.6±4.1^a^ | 7.9±5.2^a^ | 7.9±5.3^a^ |  | 0,781 | 0.032 | 0.999 |
| Stomach content (% of total) | 1.7±1.4^ab^ | 1.1±1.0^a^ | 2.0±1.9^ab^ | 2.1±1.6^ab^ | 1.9±1.6^ab^ | 1.8±1.2^ab^ | 0.9±0.6^a^ | 2.2±1.7^ab^ | 5.5±5.4^c^ | 4.0±3.6^bc^ |  | 0.732 | <0.001 | 0.289 |
| Proximal gut content (% of total) | 49.4±3.0^ab^ | 48.8±3.9^ab^ | 48.2±3.9^ab^ | 49.6±5.3^b^ | 44.8±3.0^ab^ | 43.7±4.4^a^ | 49.5±4.4^ab^ | 48.7±3.3^ab^ | 43.7±7.7^a^ | 43.1±9.7^a^ |  | 0.715 | <0.001 | 0.934 |
| Mid gut content (% of total) | 44.2±2.4^cd^ | 44.1±2.7^cd^ | 44.0±3.1^bcd^ | 40.8±3.9^bc^ | 47.2±4.4^cd^ | 48.0±4.0^d^ | 42.8±2.3^bcd^ | 42.5±2.7^bcd^ | 35.6±11.6^ab^ | 34.8±10.6^a^ |  | 0.482 | <0.001 | 0.808 |
| Distal gut content (% of total) | 4.8±1.3^a^ | 6.0±2.8^a^ | 5.8±3.7^a^ | 7.5±3.3^a^ | 6.1±2.8^a^ | 6.5±3.1^a^ | 6.9±3.7^a^ | 6.6±2.9^a^ | 15.2±13.8^ab^ | 18.1±17.4^b^ |  | 0.385 | <0.001 | 0.963 |
| Faeces score* | 2.9±0.3^a^ | 3.1±0.4^a^ | 2.9±0.6^a^ | 2.8±0.6^a^ | 2.2±0.4^a^ | 2.3±0.6^a^ | 2.4±0.5^a^ | 2.2±0.4^a^ | 2.5±0.5^a^ | 2.8±0.6^a^ |  | 0.930 | 0.292 | 0.872 |

*Score of faeces consistency as compact (dry faecal pellet; 1), solid (shaped digesta; 2), semi-solid (soft digesta; 3), runny (low viscosity digesta; 4) and diarrhoea (liquid-like; 5)

Mean ± standard deviation. Statistics by two-way ANOVA, main effect by sex and feed, followed by Tukey post hoc test. Superscript letters showing differences (*P*<0.05) between dietary groups. Data for GI content were log-transformed prior to analysis due to variance heterogeneity (Levene’s test).

**Table 4.** Picture of dissected faecal samples from distal gut, one selected sample of á 10 per tank (2 missing)

| Replicate | Control FM | Control PM | Guar Gum | Xanthan Gum | Alginate |
| --- | --- | --- | --- | --- | --- |
| 1 | 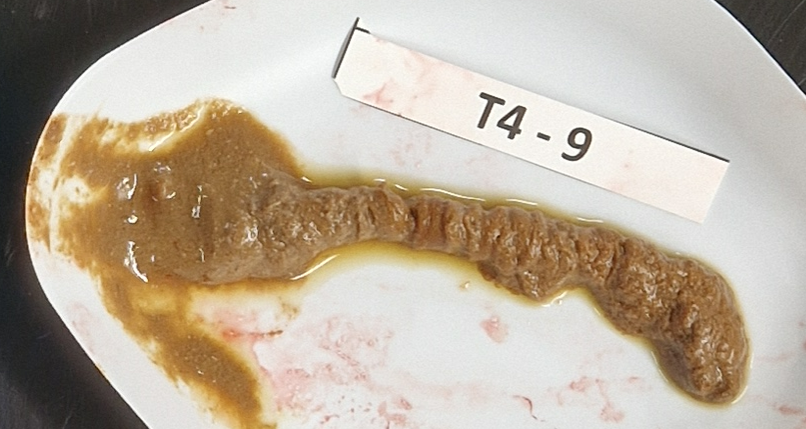 | 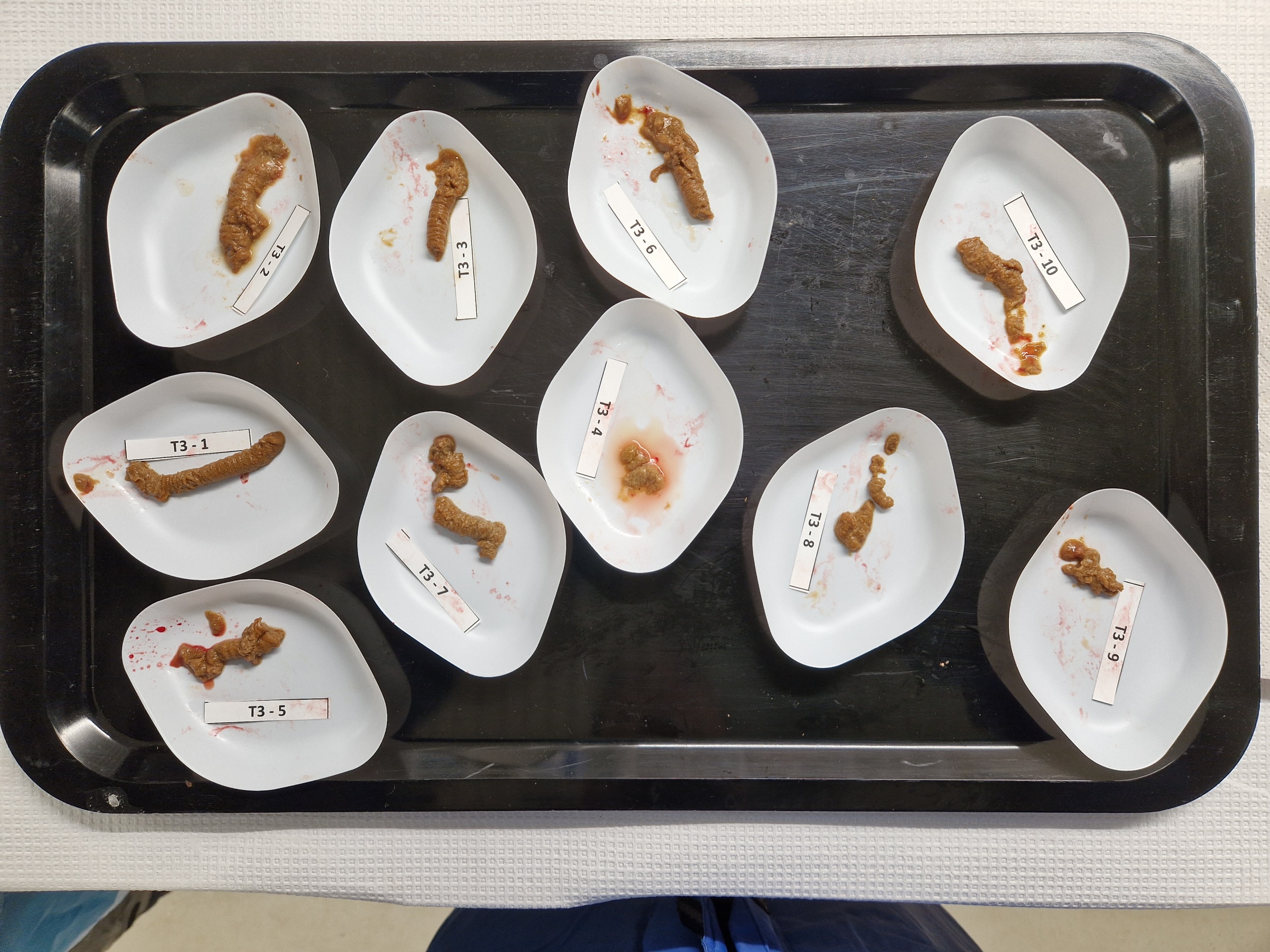 | 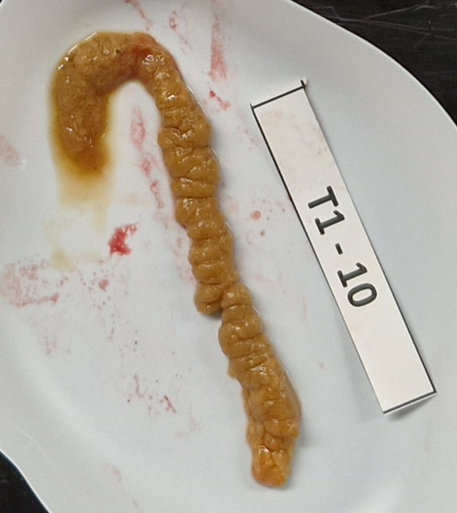 | 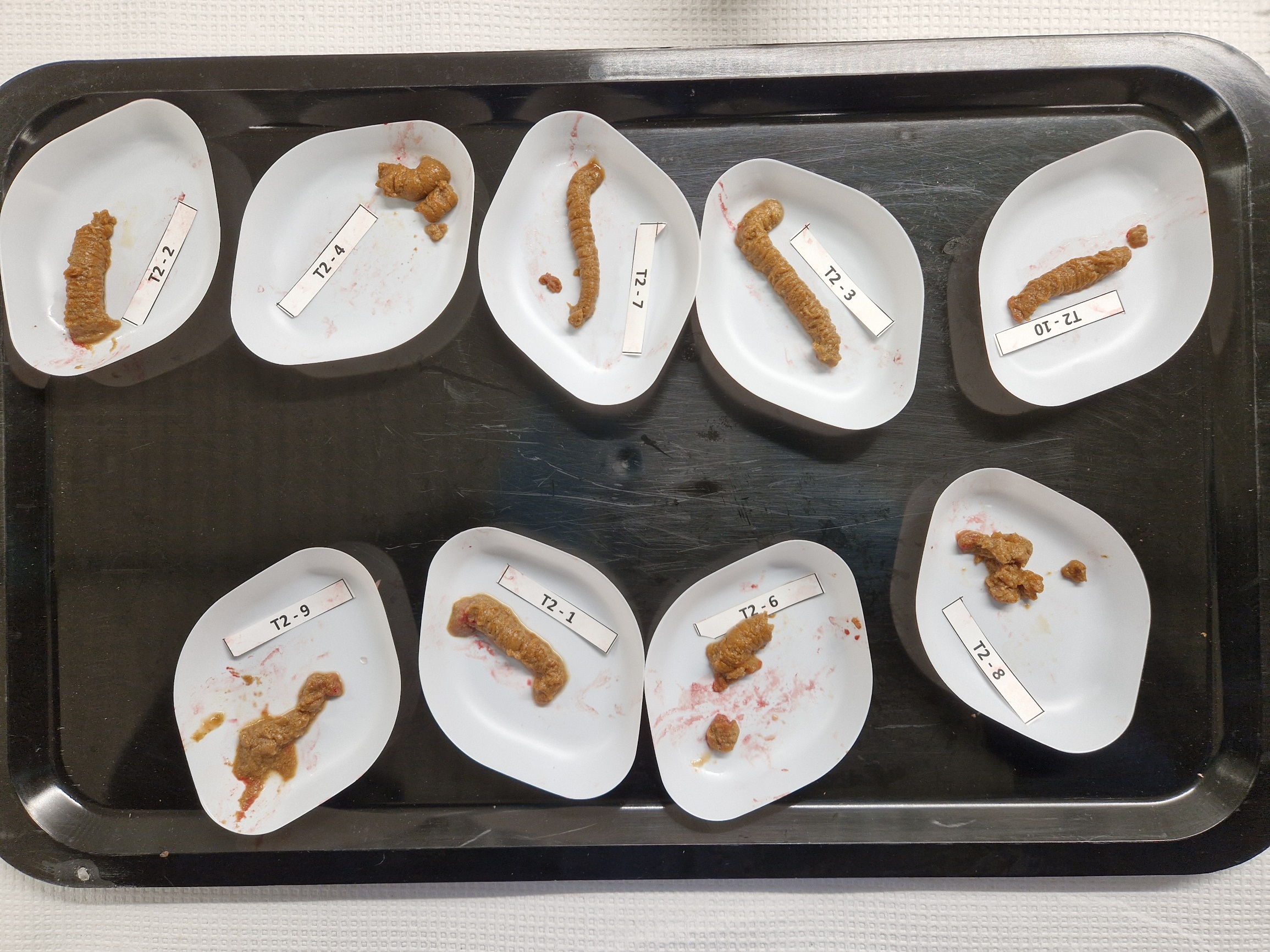 |  |
| 2 | 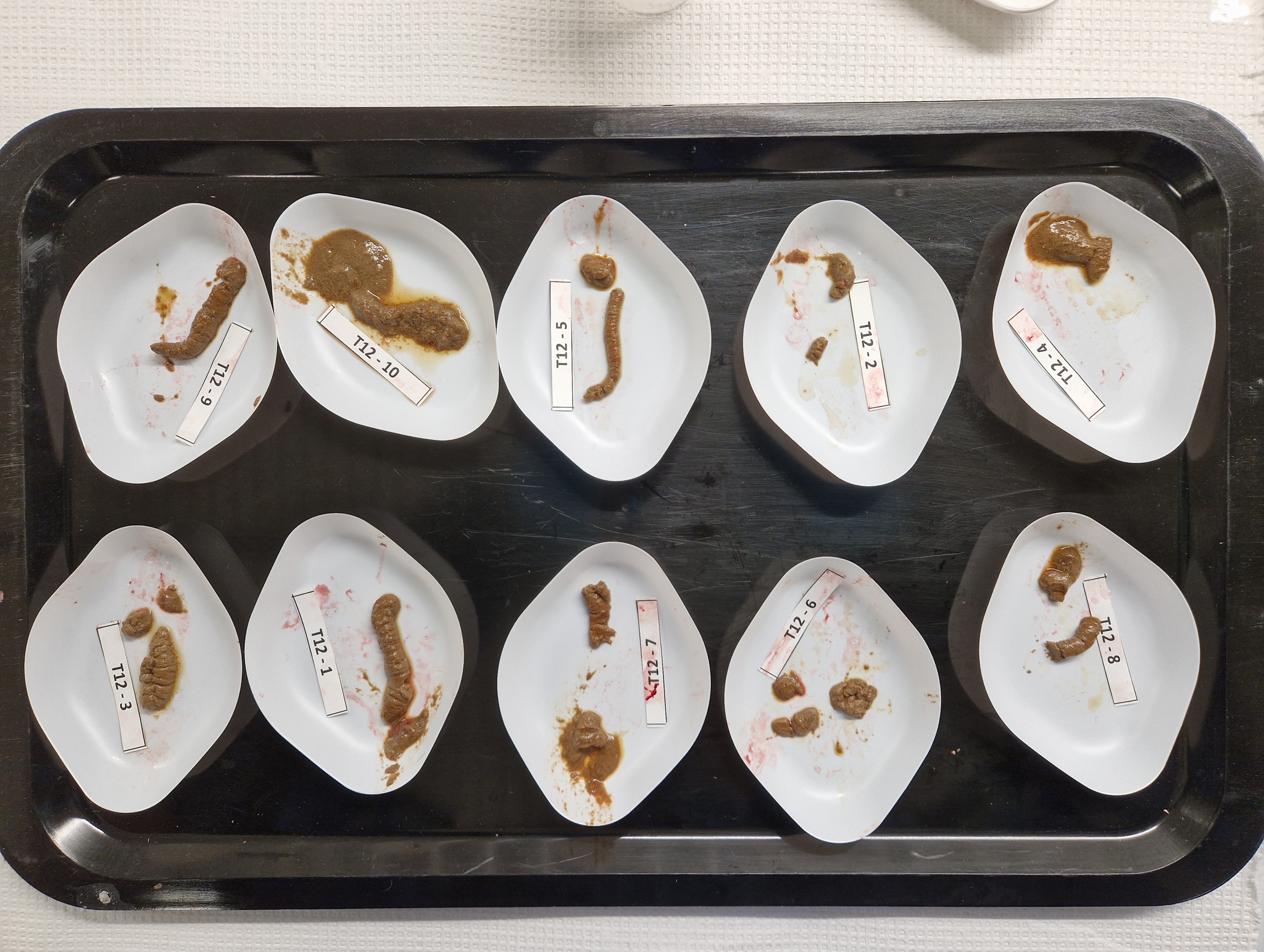 |  | 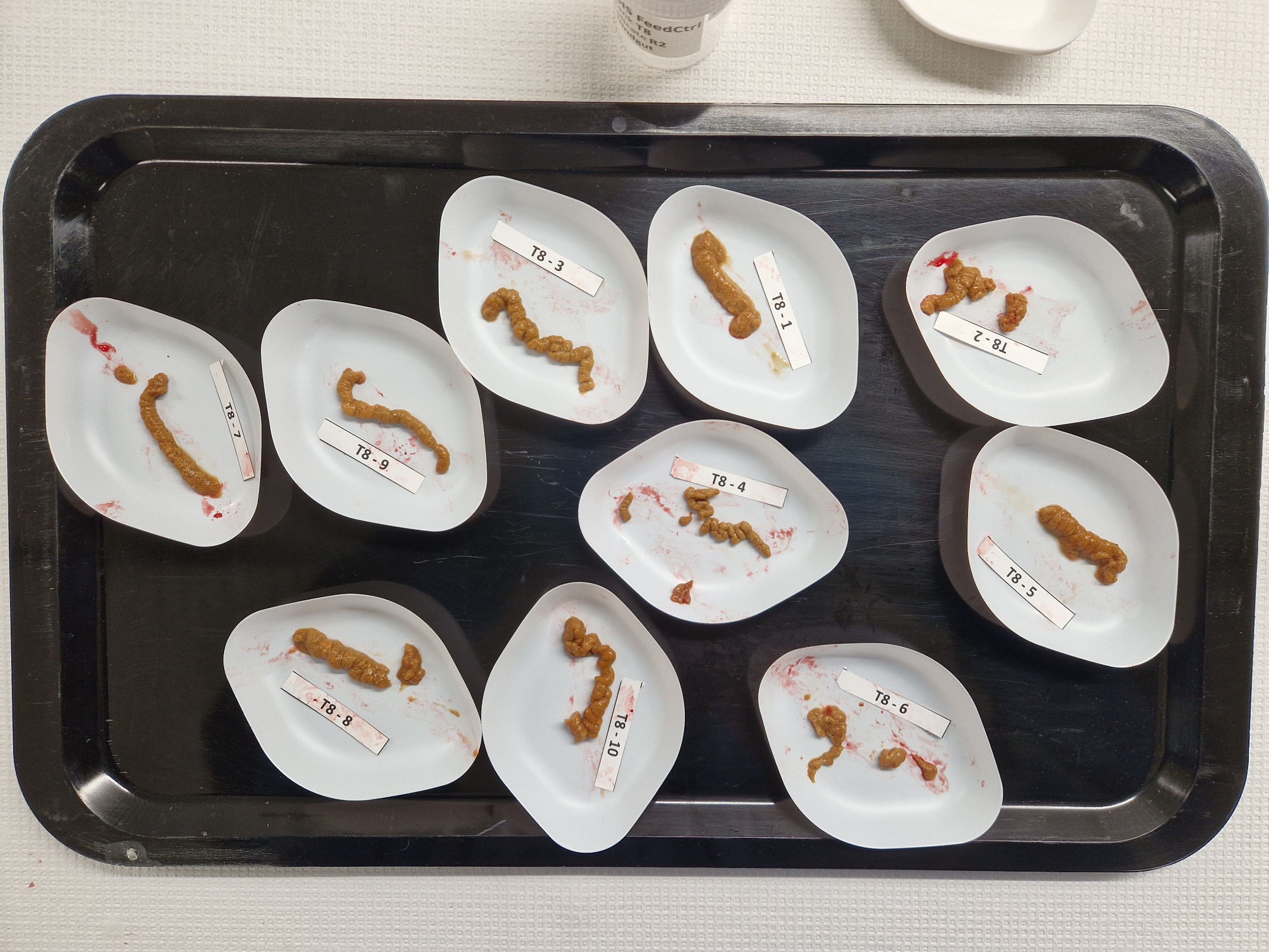 | 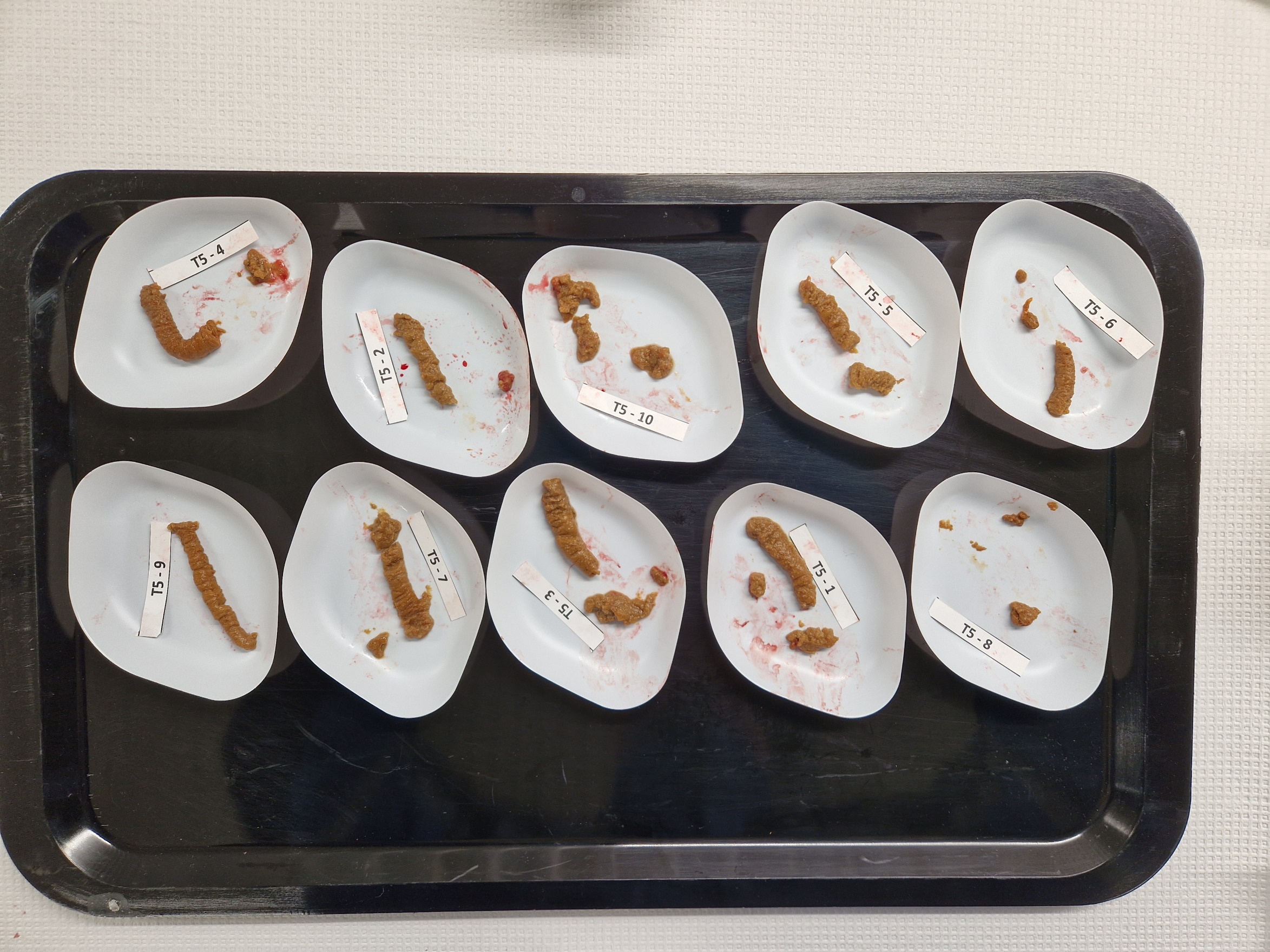 | 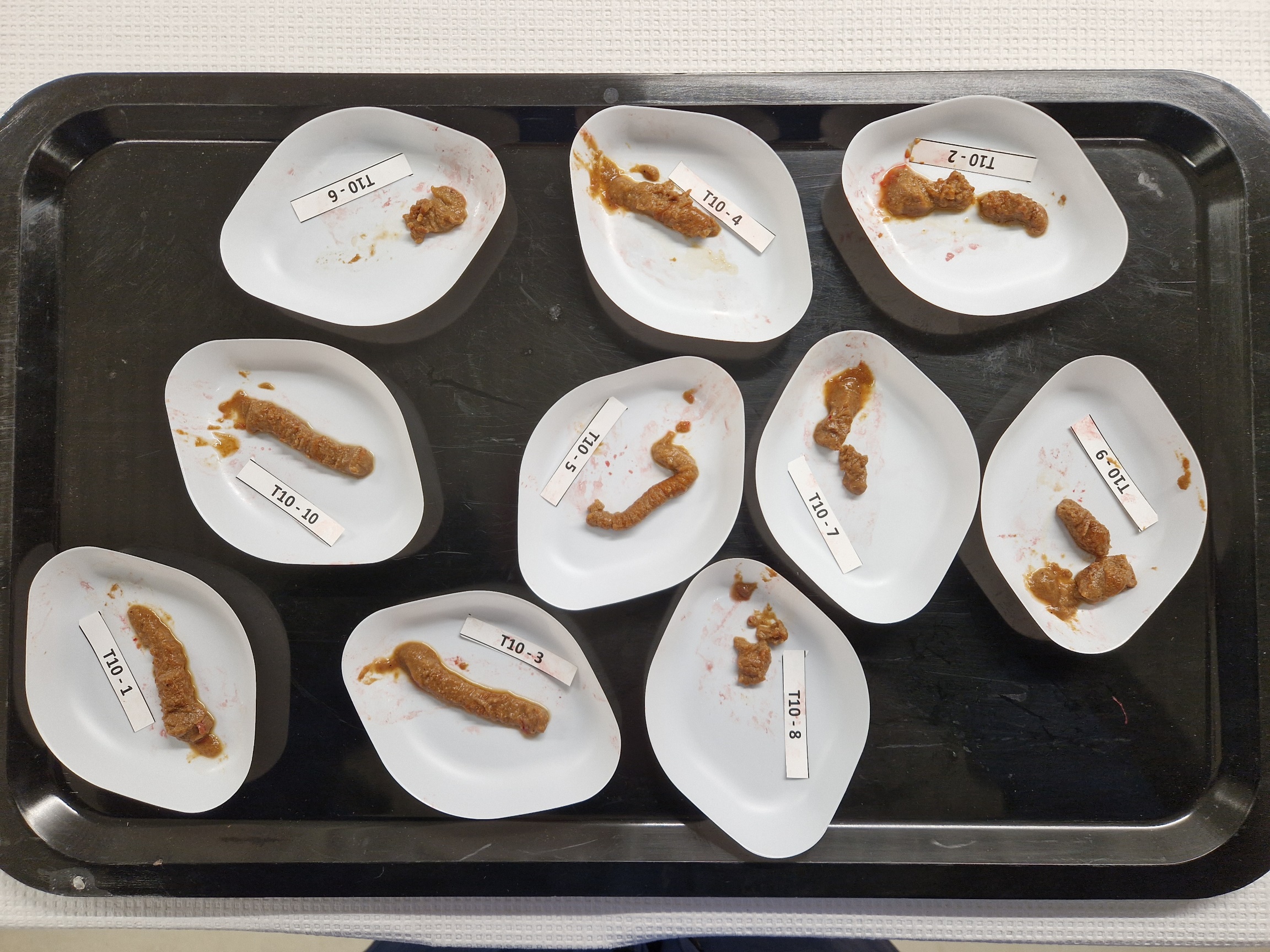 |
| 3 | 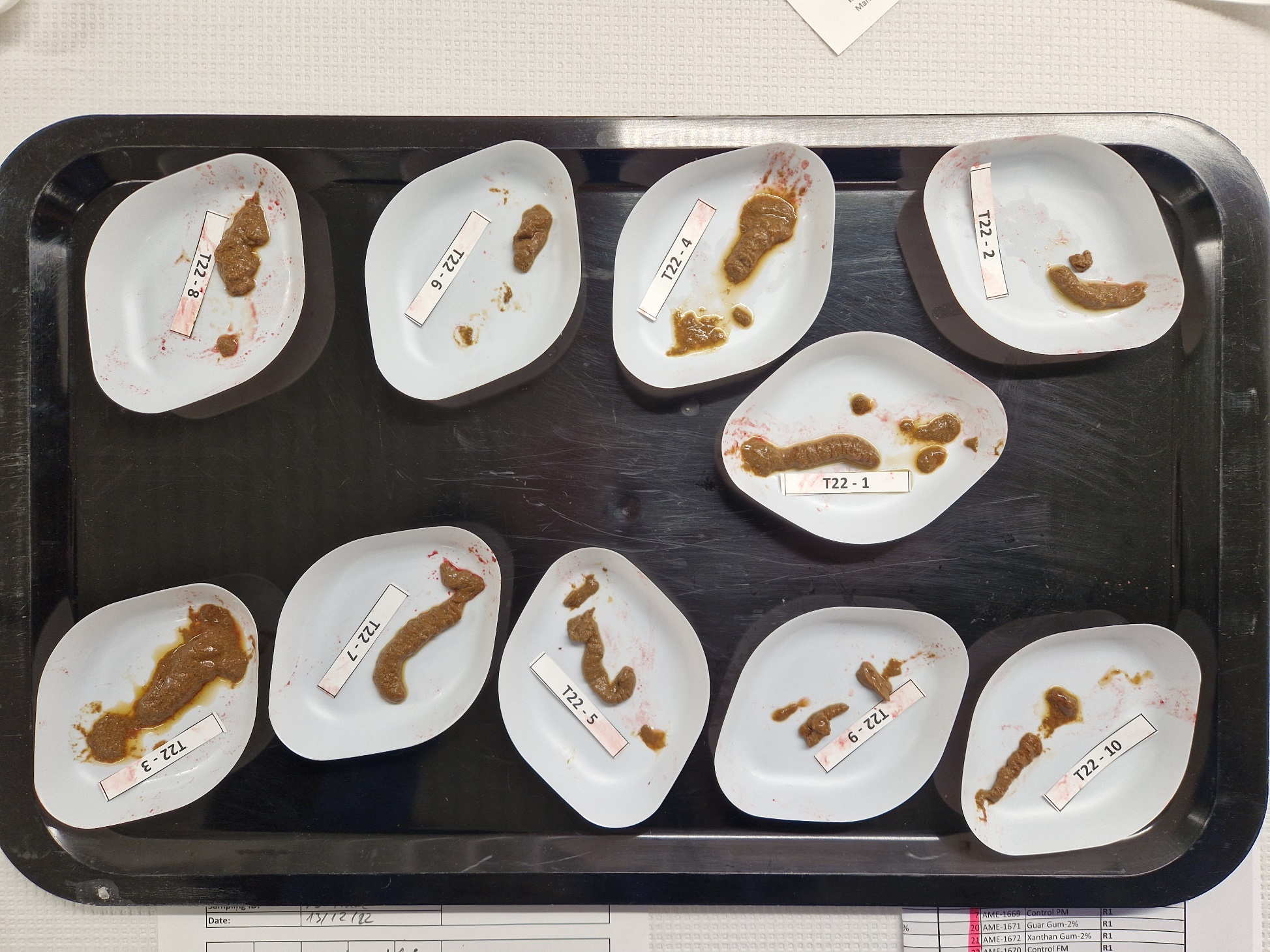 | 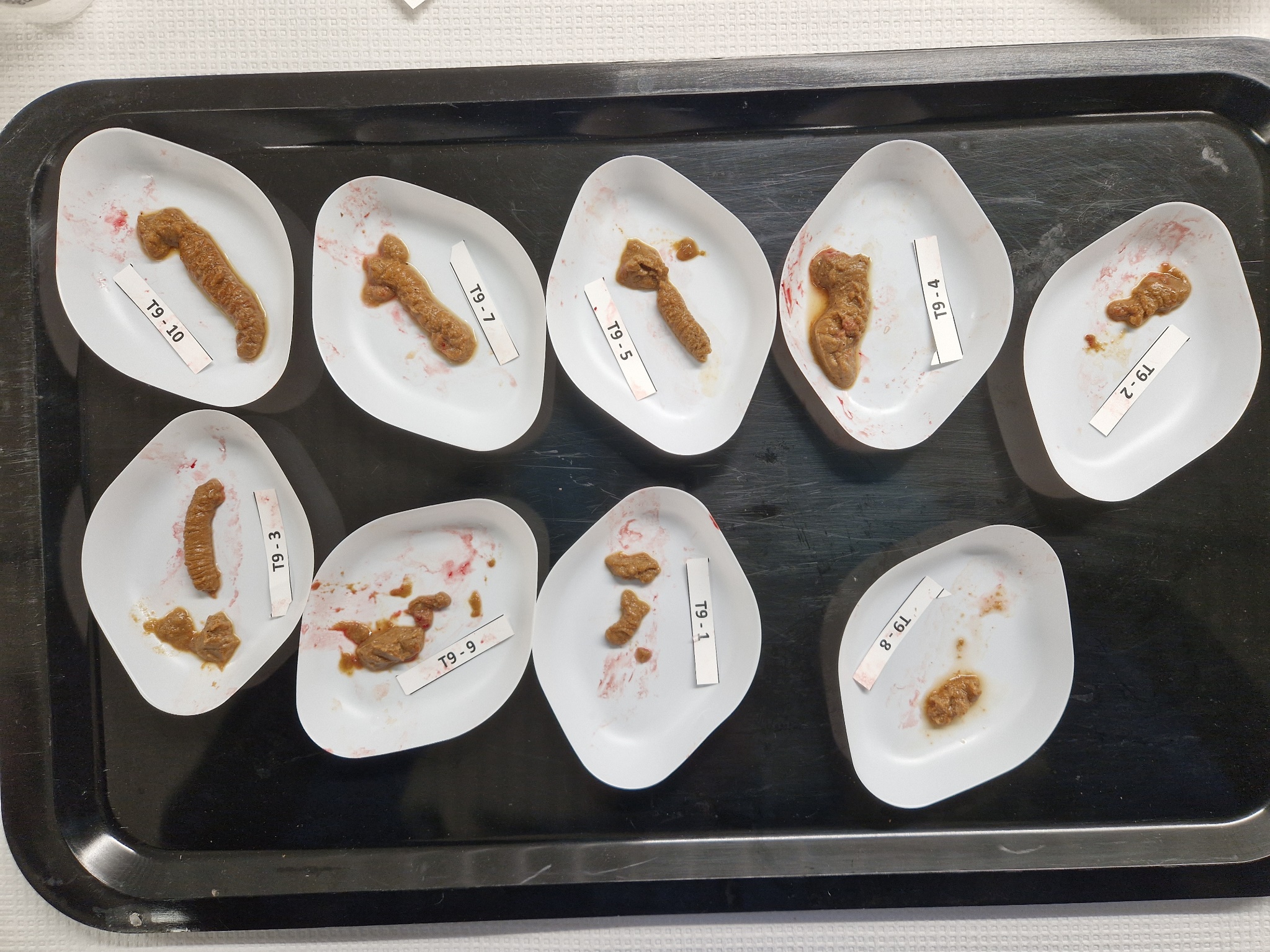 | 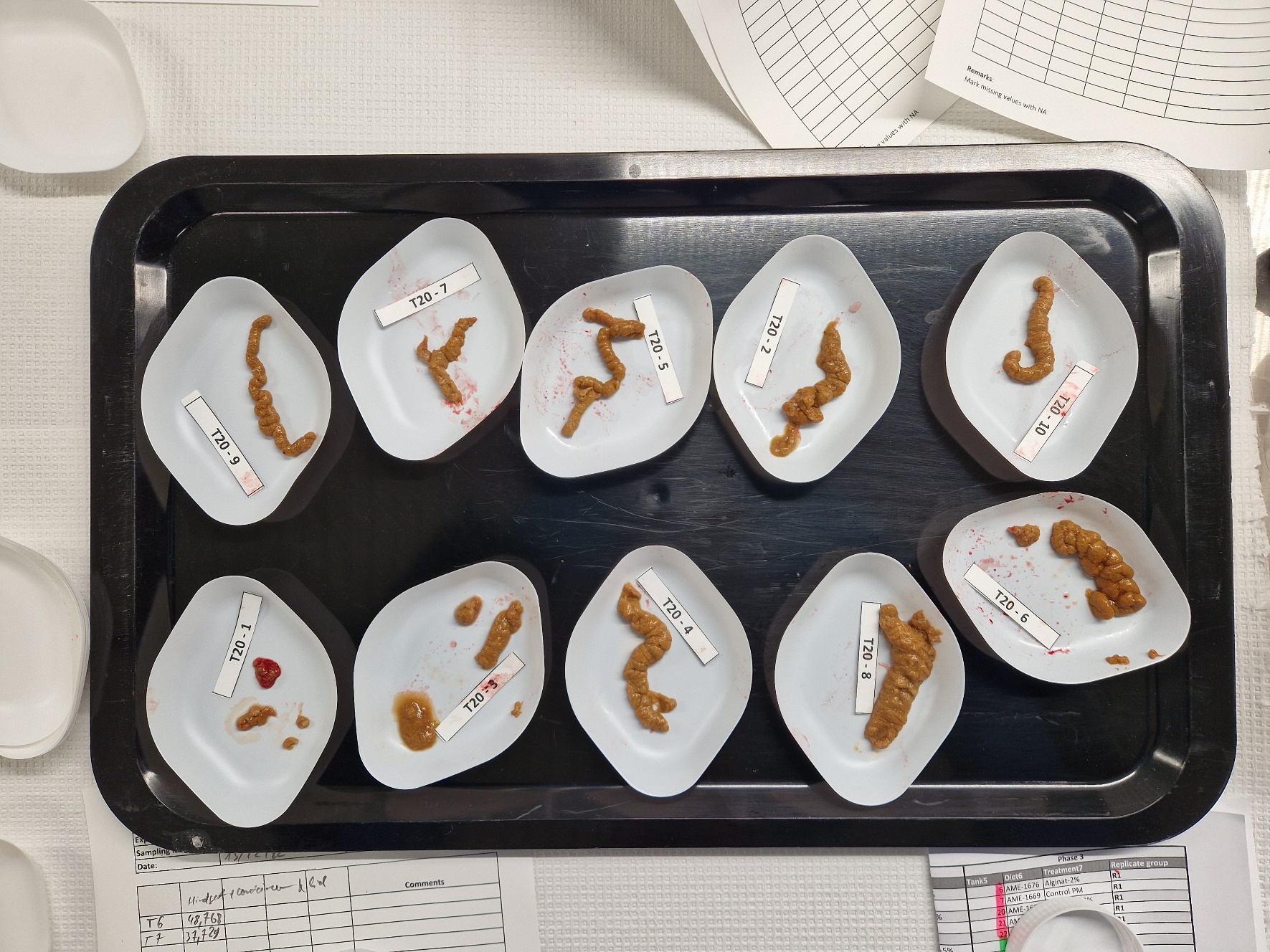 | 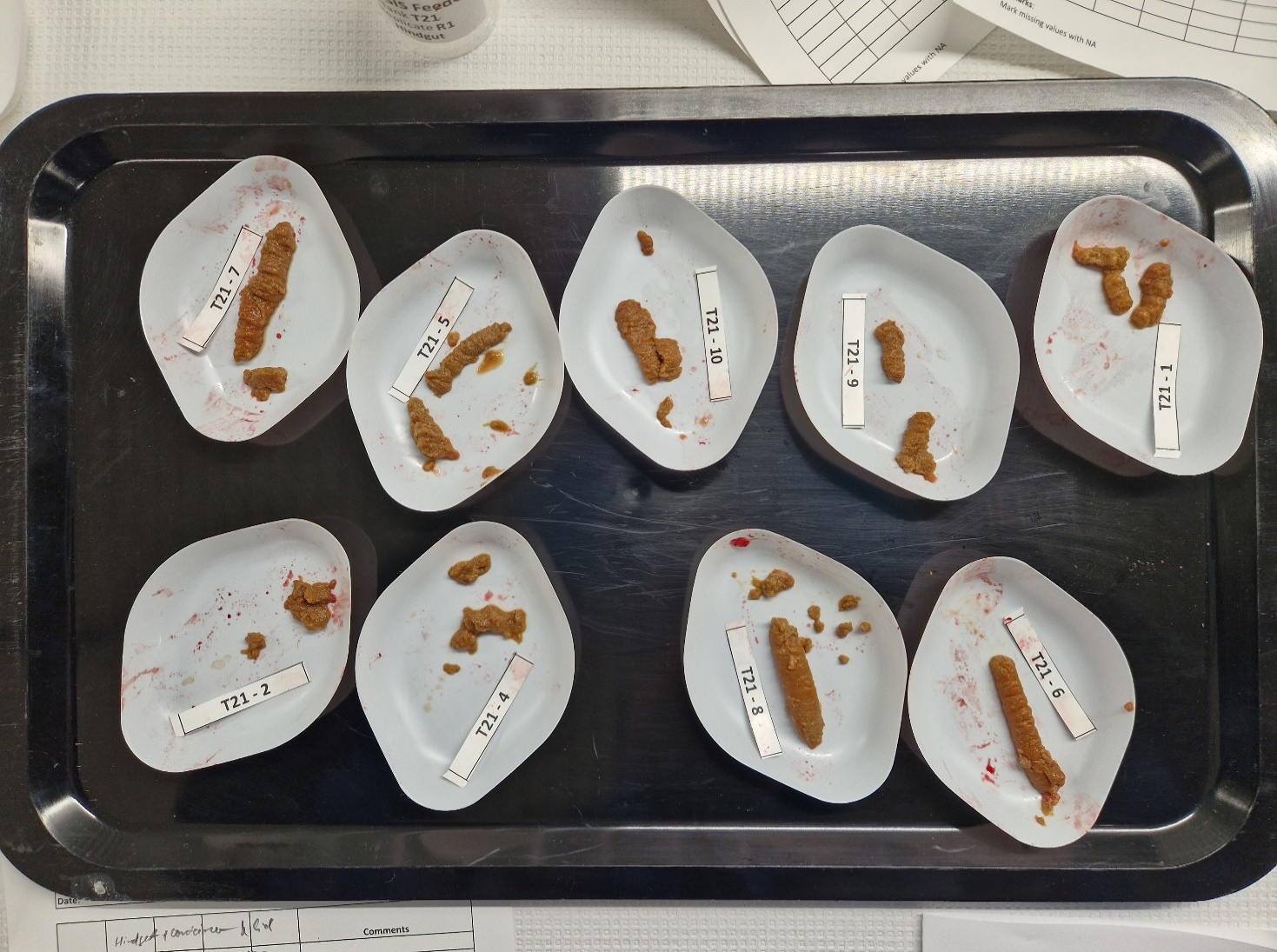 | 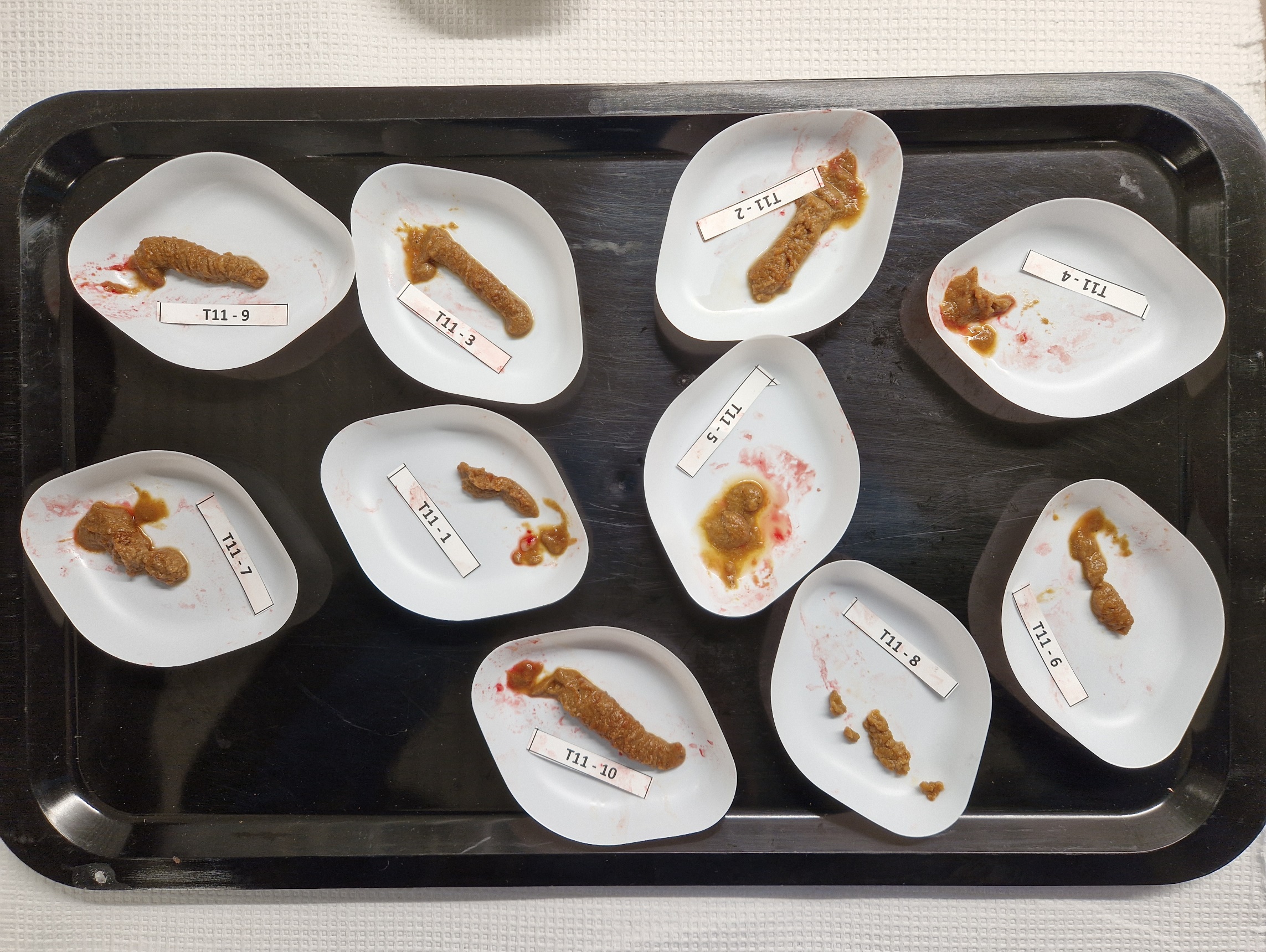 |
